# Effects of Chemogenetic Inhibition of D1 or D2 Receptor-Containing Neurons of the Substantia Nigra and Striatum in Mice With Tourette Syndrome

**DOI:** 10.3389/fnmol.2021.779436

**Published:** 2021-12-09

**Authors:** Lixue Lin, Yuye Lan, He Zhu, Lingling Yu, Shuang Wu, Wangyixuan Wan, Yang Shu, Hongchun Xiang, Tengfei Hou, Hong Zhang, Yan Ma, Wen Su, Man Li

**Affiliations:** ^1^Department of Neurobiology, School of Basic Medicine, Tongji Medical College, Huazhong University of Science and Technology, Wuhan, China; ^2^Department of Rehabilitation, Wuhan No.1 Hospital, Wuhan, China; ^3^Institute of Clinical Medicine, Zhanjiang Central People’s Hospital, Zhanjiang, China; ^4^Institute of Integrated Traditional Chinese and Western Medicine, Tongji Hospital, Tongji Medical College, Huazhong University of Science and Technology, Wuhan, China; ^5^Department of Central Laboratory, Affiliated Hospital of Jiangsu University, Zhenjiang, China; ^6^Department of Acupuncture and Moxibustion, Union Hospital, Tongji Medical College, Huazhong University of Science and Technology, Wuhan, China; ^7^Department of Pediatrics, Wuhan No.1 Hospital, Wuhan, China

**Keywords:** tourette syndrome, chemogenetic, dopamine receptors, substantia nigra pars compacta, dorsal striatum

## Abstract

As tourette syndrome (TS) is a common neurobehavioral disorder, the primary symptoms of which include behavioral stereotypies. Dysfunction of the substantia nigra–striatum network could be the main pathogenesis of TS, which is closely associated with dopamine (DA) and its receptors. TS is often resistant to conventional treatments. Therefore, it is necessary to investigate the neurobiological mechanisms underlying its pathogenesis. In this study, we investigated whether chemogenetic activation or inhibition of dopaminergic D1 receptor (D1R)- or D2 receptor (D2R)-containing neurons in the substantia nigra pars compacta (SNpc) or dorsal striatum (dSTR) affected the stereotyped behavior and motor functions of TS mice. Intraperitoneal injection of 3,3′-iminodipropionitrile (IDPN) was used to induce TS in mice. Stereotyped behavior test and open-field, rotarod, and grip strength tests were performed to evaluate stereotyped behavior and motor functions, respectively. Immunofluorescence labeling was used to detect the co-labeling of virus fluorescence and D1R or D2R. We found that chemogenetic inhibition of D1R- or D2R-containing neurons in the SNpc and dSTR alleviated behavioral stereotypies and motor functions in TS mice. Chemogenetic activation of D1R-containing neurons in the dSTR aggravated behavioral stereotypies and motor functions in vehicle-treated mice, but neither was aggravated in TS mice. In conclusion, chemogenetic inhibition of D1R- or D2R-containing neurons in the SNpc and dSTR alleviated behavioral stereotypies of TS, providing a new treatment target for TS. Moreover, the activation of D1R-containing neurons in the dSTR may contribute to the pathogenesis of TS, which can be chosen as a more precise target for treatment.

## Introduction

Tourette syndrome (TS) is a common neurobehavioral disorder, the primary symptoms of which include a variety of motor and vocal behavioral stereotypies (Hsu et al., 2021). Male patients have a higher incidence of TS than female patients, and the onset occurs before the age of 18 and lasts for more than 1 year (Ganos and Martino, 2015; Yang et al., 2016). Typically, behavioral stereotypies are defined as rapid, recurrent, and brief movements or vocalizations, the process of which gradually deteriorates (Kawohl et al., 2009). Approximately 90% of patients with TS are accompanied by neuropsychiatric diseases, among which attention-deficit hyperactivity disorder, obsessive-compulsive disorder, and impulse control disorder are the most common complications (Ganos and Martino, 2015). The process of TS reduces the quality of life in patients and puts a heavy burden on the family and society (Dodel et al., 2010; Cen et al., 2020). TS is often resistant to conventional treatments and remains a major therapeutic challenge. It is necessary to investigate the neurobiological mechanisms underlying the pathogenesis of TS and choose more precise intervention targets.

There is sufficient evidence that the basal ganglia plays an important role in the pathophysiology of TS, and the dopamine (DA) system can affect locomotion behavior. The causes of TS are mainly related to abnormalities in the substantia nigra–striatum network (Groenewegen et al., 2003; Jakubovski and Muller-Vahl, 2017; Hamamoto and Kano, 2018), which is closely associated with DA and its receptors. The dopaminergic D1 receptor (D1R) and D2 receptor (D2R) are involved in the direct and indirect pathways of basal ganglia function (Surmeier et al., 2007). Activating D1R can enhance the role of the direct pathway to promote movement, whereas activating D2R can inhibit the indirect pathway (Bromberg-Martin et al., 2010; Lobo et al., 2010; Ferguson et al., 2011). Thus, we wondered whether the manipulation of D1R- or D2R-containing neurons could affect stereotyped behavior in mice with TS, to explore the correlation between DA receptors and the neurobiological mechanism of TS.

Dopaminergic neurons in the substantia nigra pars compacta (SNpc) are the main neurons responsible for DA synthesis in the brain (Lerner et al., 2012). A large amount of DA is released in the neural circuit of SNpc projections to the dorsal striatum (dSTR) (Broderick and Phelix, 1997). In the substantia nigra–striatum network, the dSTR plays an important role in the motor regulation function mediated by the basal ganglia motor regulation pathway (Groenewegen et al., 2003; Rodrigues et al., 2017). Since the dSTR is associated with the early stages of the motor process (Kravitz et al., 2010), it may also be involved in the generation of TS-related behavioral stereotypies. The excessive release of DA in the dSTR can cause abnormal behavioral stereotypies in mice (Liu et al., 2020). After microinjection of the GABA_*A*_ antagonist picrotoxin (PTX) into the dSTR, mice showed intermittent rapid, repetitive, and non-rhythmic movements of the head and contralateral limbs, which were similar to the behavioral stereotypies of patients with TS (Pogorelov et al., 2015). Long-term administration of the D2R agonist quinpirole induced TS-related behavioral stereotypies in rats (Nespoli et al., 2018). This suggests that over-release of DA and overactivation of D2R may contribute to TS-related behavioral stereotypies. However, haloperidol, a D2R antagonist, has limited efficacy in the treatment of TS with severe extrapyramidal side effects (Silva et al., 1996; Mogwitz et al., 2013; Egolf and Coffey, 2014). Therefore, it is worthwhile to develop a new intervention method to treat TS, such as chemogenetic strategies.

Studies have shown that neural activity in the SNpc of patients with TS is stronger than that of normal subjects in the presence of spontaneous and non-spontaneous behavioral stereotypies, reflecting the overactivity of SNpc dopaminergic neurons in patients with TS (Baym et al., 2008). D1Rs and D2Rs are distributed on DA neurons in the SNpc, as well as inhibitory interneurons (Yung et al., 1995; Beaulieu and Gainetdinov, 2011; Yang et al., 2019). In addition, D2Rs are distributed on GABAergic projecting neurons of the indirect pathway and inhibitory interneurons in the dSTR (Alcantara et al., 2003; Maurice et al., 2004; Zhang et al., 2009; Jijon-Lorenzo et al., 2018; Brandenburg et al., 2020; Lewis et al., 2020). However, D1R is only expressed on GABAergic projecting neurons of the direct pathway in the dSTR (Avila-Luna et al., 2019; Assali et al., 2021), which is closely related to the regulation of dyskinesia in patients with Parkinson’s disease (Calabrese et al., 2020). Therefore, we wondered whether manipulating D1R- or D2R-containing neurons in the SNpc or dSTR by chemogenetic strategies affected behavioral stereotypies in normal and TS mice. We hypothesized that D1R in the dSTR may be a more precise target to treat TS, since it is distributed on only one type of neuron.

3,3′-Iminodipropionitrile (IDPN) is a synthetic organic nitrile, and it is the most commonly used inducer of TS in animal models as its effects can last a long time (Wang et al., 2013, 2021; Zhang et al., 2014; Zhang and Li, 2015a,b; Xie et al., 2016; Chen et al., 2019; Zhao et al., 2020; Liu et al., 2021). Our previous research results also showed that IDPN induced abnormal stereotyped behavior in mice (Lin et al., 2019), which caused a behavioral syndrome in rodents similar to the symptoms of patients with TS, such as featuring lateral and vertical head-shakes, random circling, hyperactivity, and elevated acoustic startle response (Khan and Ibrahim, 2015; Zhang and Li, 2015c; Wang et al., 2021). Therefore, we used IDPN to induce a model of TS in mice. We improved the score sheet of stereotyped behavior based on the typical symptoms of patients with TS and relevant studies, in order to evaluate the stereotyped behavior of mice (Wang et al., 2013; Proietti Onori et al., 2014).

In this study, we investigated whether chemogenetic activation or inhibition of D1R- or D2R-containing neurons in the SNpc or dSTR affected the stereotyped behavior and motor functions of IDPN-induced TS mice, in order to find the neurobiological mechanism of the pathogenesis of TS and choose more precise intervention targets for the treatment of TS.

## Materials and Methods

### Animals

All D1R-cre mice [MMRRC Tg (Drd1a cre) EY262Gsat], D2R-cre mice [MMRRC Tg (Drd2-cre) ER44Gsat], and their wild-type littermate (WT) mice (male, aged 8 weeks, and 18–21g) maintained on a C57BL/6 congenic background were kindly provided by Professor Tonghui Xu (Fudan University, Shanghai, China) (Li et al., 2018). All animal experimental protocols conformed to the Animal Management Rules of the Chinese Ministry of Health, and this study was approved by the Animal Ethics Committee of the Chinese Academy of Medical Sciences. The mice were maintained in a controlled environment with a temperature of 21 ± 1°C and a relative humidity of 60% ± 10% under a 12-h light/dark cycle (lights on at 7 a.m.), and had free access to food and water. The mice were housed individually in standard polypropylene plastic cages (318 mm × 202 mm × 136 mm) with sawdust bedding and water and food *ad libitum* (Xietong Pharmaceutical Biotechnology Limited Liability Company, Jiangsu, China).

### Viruses Constructs and Surgery

Chemogenetic strategies: rAAV-hSyn-DIO-hM3D(Gq)-mCherry-WPRE-pA (hM3Dq) or rAAV-hSyn-DIO-hM4D(Gi)-mCherry-WPREs-pA (hM4Di) were microinjected into the bilateral SNpc or dSTR, respectively, of D1R-cre, D2R-cre, or wild-type littermate mice, to activate or inhibit neurons containing dopaminergic D1R and D2R. rAAV-hSyn-DIO-mCherry was selected as the control virus. All viruses used in this study were acquired from Wuhan BrainVTA Scientific and Technical Corporation.

Before surgery, each mouse was anesthetized with isoflurane and fixed in a stereotaxic apparatus (RWD Instruments, China). A 1.5-cm-long longitudinal incision was made along the midline of the skull, and the periosteum was gently removed from the exposed surface of the surgical area. The coordinates of the SNpc were as follows: -3.3 mm from the bregma, 1.6 mm lateral from the midline, and 3.7 mm ventral to the skull. The coordinates of the dSTR were as follows: 0.5 mm from the bregma, 1.5 mm lateral from the midline, and 2.7 mm ventral to the skull. Desired virus vectors (200 nL) were injected into the SNpc or dSTR at a rate of 30 nL per 60 s.

### Tourette Syndrome Model

The adaptation period between arrival at the laboratory and the start of testing was 1 week. After 1 week, the vehicle group mice were intraperitoneally (i.p.) injected with saline (0.9%) once a day for 7 consecutive days. The TS group mice were intraperitoneally injected with IDPN (350 mg/kg, Sigma, St. Louis, MO, United States) once a day for 7 consecutive days. The ethological score between each group was balanced, by referring to the evaluation grade of stereotypy (Table 1). On the 8th day, the score of stereotyped behavior of mice was greater than or equal to 2 points, which proved a successful IDPN-induced TS model.

**TABLE 1:** Behavior measurements referring to evaluating grades of stereotypy.

Score	Stereotypy
0	No stereotypy or normal activity^a^
1	Discontinuous circling behavior^b^ Occasional head twitching
2	Occasionally vertical dyskinetic head and neck movements Occasional sniffing, licking, and biting
3	Continuous circling behavior, increased body raising Increased sniffing, repetitive grooming (such as paw-to-mouth movements)^c^
4	Increased lateral and vertical dyskinetic head and neck movements

*^a^Such as immobility (no visible movement of the animal).*

*^b^Such as clockwise/counterclockwise circling, loosely circular path traced on the cage floor.*

*^c^Such as head-down sniffing (nose in contact with the floor), grooming (self-cleaning with mouth or paws), and face washing (forepaws moving back and forth from the ears to the snout and mouth).*

### Behavioral Tests

#### Stereotyped Behavior Test

The stereotyped behavior test was conducted by two trained and independent observers who were familiar with the measurements but blinded to the group allocation. They grouped the mice themselves and then randomly tested all mice and scored them according to the random number table method. Finally, the results were correctly counted by an experimenter who was aware of the group allocation. The observers placed the mice in the box and recorded a 30-min video for each mouse. The apparatus used for stereotyped behavior was a round, black plastic box with a diameter of 16 cm and a height of 14 cm, and the camera was placed at the top. The light condition during the observation period was created with an LED tube (consistent with the illumination of the usual living environment of the mouse), which lasted throughout the experiment. Before the test, the observers sprayed the inside and bottom of the box and the transparent lid with 75% alcohol and wiped it with a paper towel. Each animal was observed for 1 min every 5 min for a total of six periods. One or more episodes in accordance with the grades were used to obtain the corresponding score and calculate the average score on the basis of results from two observers, as objective indicators of behavioral changes (Table 1).

#### Rotarod Test

To assess the motor coordination of mice, they were individually placed on a rotarod (YLS-4C, Yiyan, Jiang, China) for 5 min at 4 rpm at 10:00 a.m. and 5:00 p.m. for 3 days before the formal test, to allow the mice to learn how to use the apparatus. The rotarod moved at an initial speed of 4 rpm and subsequently accelerated to 40 rpm in 5 min. Mice were held by the tail and placed on the rotarod, facing away from the direction of rotation. The falling time after the acceleration began was recorded. Each mouse received three test sessions with at least a 5-min interval between them, and the average time was calculated.

#### Grip Strength Test

To evaluate the muscle strength of mice, we performed a grip strength test. We followed the manufacturer’s instructions for the grip strength meter (YLS-13A, Zhenghua, Anhui, China), and the statistical indicator was the grip force (g). Mice were placed on a base plate (230 mm × 250 mm) in front of a grasping bar. The bar was fitted to a force transducer connected to a peak amplifier. The mice were pulled with their tails when grasping the bar. The maximal grip force was measured within 20 s.

#### Open-Field Test

We used a 50 cm × 50 cm black-walled open-field test box, and a camera was installed directly above to record the trajectory. After each mouse was tested, 75% alcohol was sprayed on the test area and wiped with a paper towel, and then the next mouse was placed. During the test, the mice were allowed to move freely in the test area. The test duration for each mouse was 10 min. This study recorded the movement trajectory, total distance (mm), and resting time (s) of each mouse. The data were processed and analyzed using the SuperMaze software (Xinsoft SuperMaze Animal Behavior Analysis System, Shanghai, China).

### Immunofluorescence Labeling

On the 24th day, the mice of D1R-cre + Vehicle + Gi + i.p. Saline, D1R-cre + Vehicle + Gq + i.p. Saline, D2R-cre + Vehicle + Gi + i.p. Saline, and D2R-cre + Vehicle + Gq + i.p. Saline group (*n* = 6 in each) were deeply anesthetized with 1% sodium pentobarbital anesthesia (50 mg/kg, i.p.) and transcardially perfused with 100 mL of 37°C normal saline followed by 50 mL of 4% paraformaldehyde in 0.1 M phosphate buffered saline (PBS, pH 7.4) at 4°C for fixation. The brain tissues were quickly separated and post-fixed for 6–8 h in the same fixative solution and dehydrated in 20% sucrose in 0.1 M PBS for 24 h and 30% sucrose in 0.1 M PBS for 24 h at 4°C. The sections were cut (60 μm in thickness) on a cryostat, mounted onto gelatin-coated slides, and air-dried overnight.

The sections were rinsed in 0.01 M PBS, blocked for 1 h with 5% donkey serum and 0.2% Tween-20 in PBS, and then incubated with the following primary antibodies at 37°C for 1 h and at 4°C overnight: rabbit anti-D1R (1:500, Ab20066, Abcam) and rabbit anti-D2R (1:100, PAA673Mu01, Cloud-Clone). Subsequently, the sections were washed four times in PBS for 5 min and incubated with the corresponding secondary antibodies, donkey anti-rabbit IgG conjugated with DyLight 488 (1:400, 711-545-152, Jackson Immune Research). Sections were washed four times with 0.05% Tween-20 in PBS for 5 min and then cover-slipped. Olympus BX51 fluorescence microscope was used to view the sections, and images were captured using Qimaging Camera and QCapture software. Five to six sections were randomly selected from each mouse. Images were analyzed using the NIH Image J software (Bethesda, MD, United States). The layouts of the images were based on Photoshop CS5 (ADOBE Company, United States).

### Data Analysis

All data analyses were conducted using GraphPad Prism 7 (GraphPad Software, Inc., La Jolla, CA, United States). Data are presented as the mean ± SEM. Normality of data was checked by Shapiro–Wilk test. We used one-way ANOVA and Tukey’s *post hoc* test to analyze behavioral data between different groups. *P* < 0.05 was considered statistically significant.

## Results

### The Neurons Containing Dopaminergic D1 Receptor or Dopaminergic D2 Receptor in the Substantia Nigra Pars Compacta or Dorsal Striatum Were Effectively Transfected With hM4Di or hM3Dq

**FIGURE 1:**
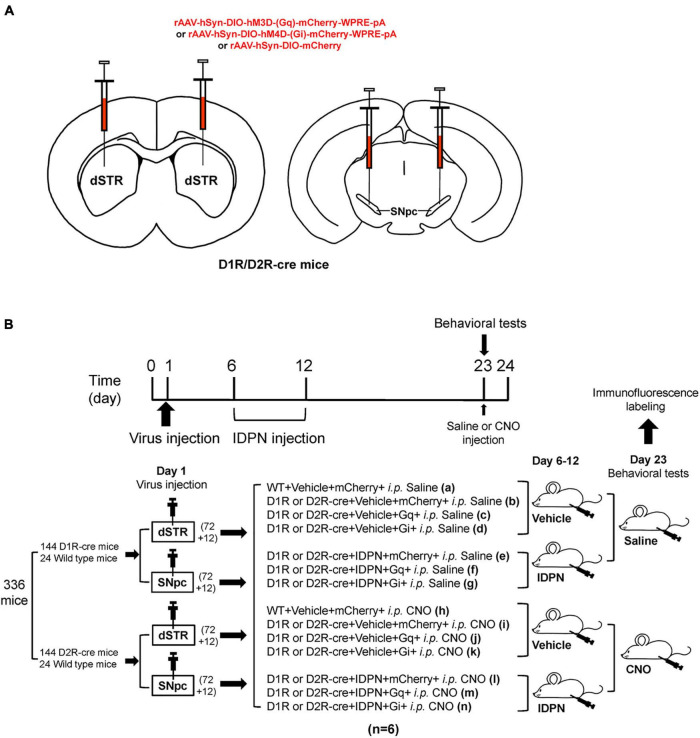
Experimental design. **(A)** Schematic diagram of the virus injection area. **(B)** Experimental design timeline. A total of 336 mice [including 144 D1R-cre mice and 24 wild-type littermate (WT) mice, 144 D2R-cre mice, and 24 WT mice] participated in this experiment. After adaptive feeding for 1 week, they were randomly divided into four large groups (84 mice in each large group), among which 72 D1R-cre mice and 12 WT mice were further divided into two small groups, and 72 D2R-cre mice and 12 WT littermate mice were further divided into two small groups. On the first day, one small group of D1R-cre mice and WT littermate mice, one small group of D2R-cre mice and WT mice were injected with the virus in the bilateral dSTR (200 nL/side), the other two small groups were injected with the virus in the bilateral SNpc (200 nL/side). Eighty-four mice in each large group were randomly divided into 14 groups according to the injections of different drugs or virus, including WT + Vehicle + mCherry + i.p. Saline (group a), D1R or D2R-cre + Vehicle + mCherry + i.p. Saline group (group b), D1R or D2R-cre + Vehicle + Gq + i.p. Saline group (group c), D1R or D2R-cre + Vehicle + Gi + i.p. Saline group (group d), D1R or D2R-cre + IDPN + mCherry + i.p. Saline group (group e), D1R or D2R-cre + IDPN + Gq + i.p. Saline group (group f), D1R or D2R-cre + IDPN + Gi + i.p. Saline group (group g), WT + Vehicle + mCherry + i.p. CNO group (group h), D1R or D2R-cre + Vehicle + mCherry + i.p. CNO group (group i), D1R or D2R-cre + Vehicle + Gq + i.p. CNO group (group j), D1R or D2R-cre + Vehicle + Gi + i.p. CNO group (group k), D1R or D2R-cre + IDPN + mCherry + i.p. CNO group (group l), D1R or D2R-cre + IDPN + Gq + i.p. CNO group (group m) and D1R or D2R-cre + IDPN + Gi + i.p. CNO group (group n) (*n* = 6 mice in each group). Mice in the groups a, b, e, h, i, and l were injected with a control virus (mCherry). Mice in the groups c, f, j, and m were injected with hM3Dq. Mice in group d, g, k, and n were injected with hM4Di. After the virus injection, all mice were back to the cage to rest for 4 days (days 2–5). On days 6–12, mice in groups e, f, g, l, m, and n were intraperitoneally injected with IDPN at 10:00 a.m. once daily, and mice in the other groups were intraperitoneally injected with 0.9% saline (Vehicle, contrast with IDPN). On the 23rd day, mice in the a–g groups were intraperitoneally injected with 0.9% saline (contrast with CNO), and mice in the h–n groups were intraperitoneally injected with clozapine N-oxide (CNO, Sigma, St. Louis, MO, United States, 1 mg/kg). Thirty minutes later, behavioral tests were carried out to compare the differences between the groups. Brain tissues for immunofluorescence labeled were collected on day 24.

In order to investigate the correlation between the neurons containing D1R or D2R in the SNpc or dSTR and locomotor activities in TS mice, we used D1R-cre, D2R-cre, and their wild-type littermate (WT) mice. The hM3Dq, hM4Di, or control virus was microinjected into the SNpc or dSTR (Figure 1A). Then, IDPN was injected intraperitoneally to establish the TS model. After the virus was fully expressed for 3 weeks, behavioral tests were performed (Figure 1B).

Immunofluorescence labeling showed that there were co-labels of hM4Di and D1R or D2R, and hM3Dq and D1R or D2R (total co-labeling rate was greater than 90%) in the SNpc (Supplementary Figure 1) and dSTR (Supplementary Figure 2), respectively, indicating that the microinjection of the virus effectively transfected neurons containing D1R or D2R in the SNpc and dSTR.

### Chemogenetic Inhibition of Dopaminergic D1 Receptor-Containing Neurons in the Substantia Nigra Pars Compacta Significantly Alleviated Involuntary Behavioral Stereotypies of Tourette Syndrome Mice

**FIGURE 2:**
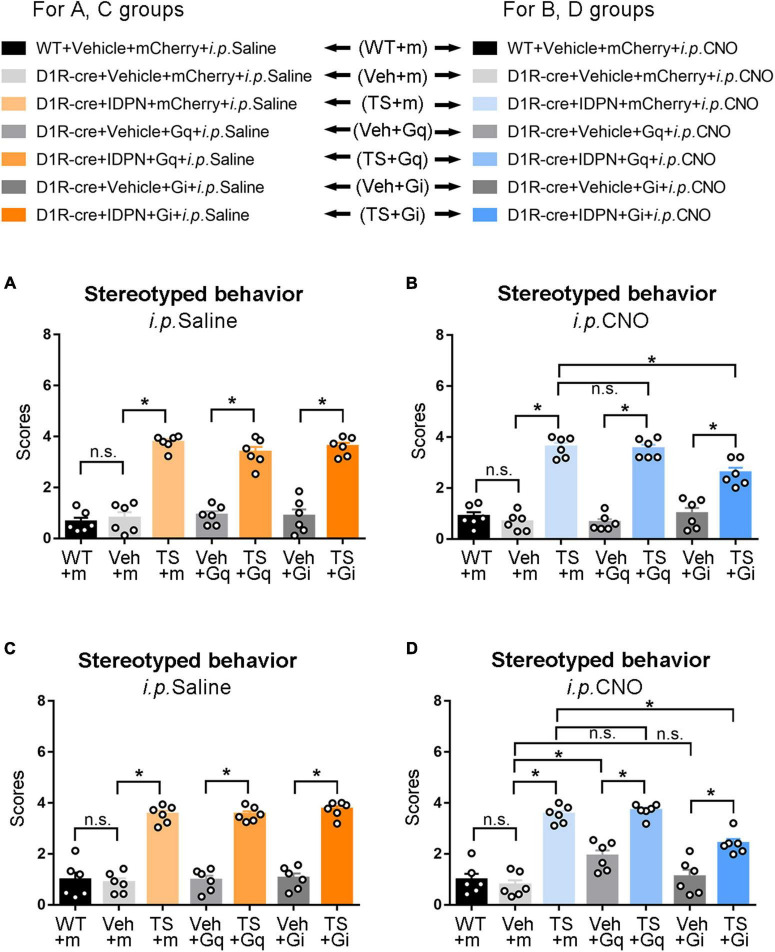
Effects of activation or inhibition of the D1R-containing neurons in the SNpc and dSTR on stereotyped behavior in mice. **(A)** Evaluations of stereotyped behavior scores of mice injected with virus in the SNpc 30 min after the injection of saline (i.p. Saline) in the WT + Vehicle + mCherry + i.p. Saline group (WT + m), D1R-cre + Vehicle + mCherry + i.p. Saline group (Veh + m), D1R-cre + IDPN + mCherry + i.p. Saline group (TS + m), D1R-cre + Vehicle + Gq + i.p. Saline group (Veh + Gq), D1R-cre + IDPN + Gq + i.p. Saline group (TS + Gq), D1R-cre + Vehicle + Gi + i.p. Saline group (Veh + Gi), D1R-cre + IDPN + Gi + i.p. Saline group (TS + Gi) on day 23. **(B)** Evaluations of stereotyped behavior scores of mice injected with virus in the SNpc 30 min after the injection of CNO (i.p. CNO) in the WT + Vehicle + mCherry + i.p. CNO group (WT + m), D1R-cre + Vehicle + mCherry + i.p. CNO group (Veh + m), D1R-cre + IDPN + mCherry + i.p. CNO group (TS + m), D1R-cre + Vehicle + Gq + i.p. CNO group (Veh + Gq), D1R-cre + IDPN + Gq + i.p. CNO group (TS + Gq), D1R-cre + Vehicle + Gi + i.p. CNO group (Veh + Gi), D1R-cre + IDPN + Gi + i.p. CNO group (TS + Gi) on day 23. **(C)** Evaluations of stereotyped behavior scores of mice injected with virus in the dSTR 30 min after the injection of saline (i.p. Saline) in each group on day 23. **(D)** Evaluations of stereotyped behavior scores of mice injected with virus in the dSTR 30 min after the injection of CNO (i.p. CNO) in each group on day 23. Data are expressed as mean ± SEM (*n* = 6 mice in each group), and the black line segment indicates the differences between groups, * represents *P* < 0.05 between marked groups, n.s. represents *P* > 0.05 between marked groups.

**FIGURE 3:**
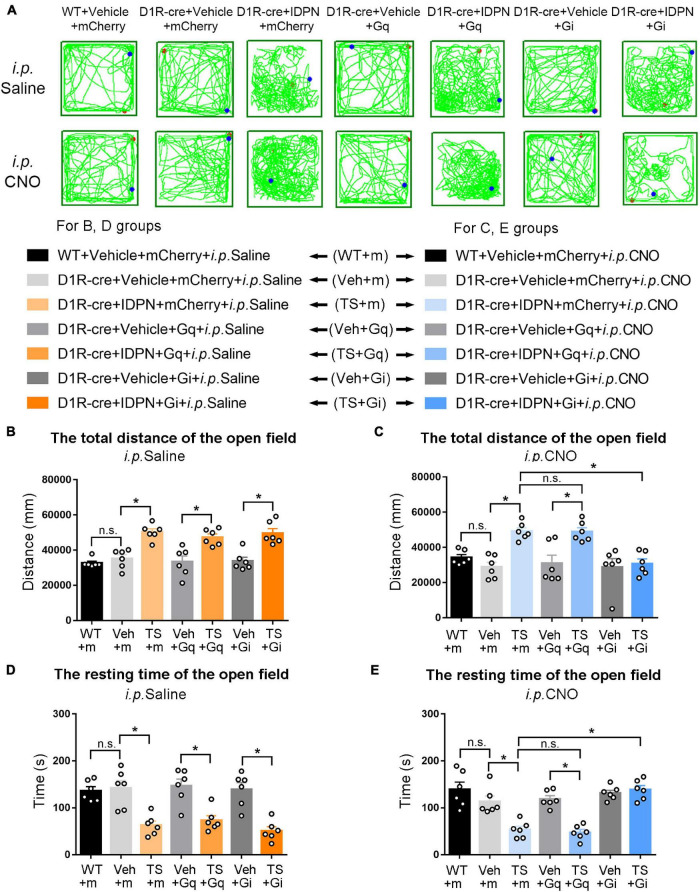
Effects of activation or inhibition of D1R-containing neurons in the SNpc on total distance and resting time in the open-field test in mice. **(A)** Evaluations of the trajectory in the open-field test in mice 30 min after the injection of saline (i.p. Saline) or CNO (i.p. CNO) in the WT + Vehicle + mCherry + i.p. Saline group (WT + m), D1R-cre + Vehicle + mCherry + i.p. Saline group (Veh + m), D1R-cre + IDPN + mCherry + i.p. Saline group (TS + m), D1R-cre + Vehicle + Gq + i.p. Saline group (Veh + Gq), D1R-cre + IDPN + Gq + i.p. Saline group (TS + Gq), D1R-cre + Vehicle + Gi + i.p. Saline group (Veh + Gi), D1R-cre + IDPN + Gi + i.p. Saline group (TS + Gi), WT + Vehicle + mCherry + i.p. CNO group (WT + m), D1R-cre + Vehicle + mCherry + i.p. CNO group (Veh + m), D1R-cre + IDPN + mCherry + i.p. CNO group (TS + m), D1R-cre + Vehicle + Gq + i.p. CNO group (Veh + Gq), D1R-cre + IDPN + Gq + i.p. CNO group (TS + Gq), D1R-cre + Vehicle + Gi + i.p. CNO group (Veh + Gi), D1R-cre + IDPN + Gi + i.p. CNO group (TS + Gi) on day 23. **(B)** Total distance in the open-field test within 10 min in mice of each group 30 min after the injection of saline (i.p. Saline) on day 23. **(C)** Total distance in the open-field test within 10 min in mice of each group 30 min after the injection of CNO (i.p. CNO) on day 23. **(D)** Resting time in the open-field test within 10 min in mice of each group 30 min after the injection of saline (i.p. Saline) on day 23. **(E)** Resting time in the open-field test within 10 min in mice of each group 30 min after the injection of CNO (i.p. CNO) on day 23. Data are expressed as mean ± SEM (*n* = 6 mice in each group), and the black line segment indicates the differences between groups, * represents *P* < 0.05 between marked groups, and n.s. represents *P* > 0.05 between marked groups.

Behavioral tests were performed to explore the changes in locomotor activity in mice after D1R-containing neurons in the SNpc were activated or inhibited by the chemogenetic approach. We found that after injection of saline (i.p. Saline, contrast with CNO), there was no significant difference in the score of stereotyped behavior, total distance, resting time in the open-field test, retention time on the rotarod, and grip force between the WT + Vehicle + mCherry + i.p. Saline group (WT + m) and D1R-cre + Vehicle + mCherry + i.p. Saline group (Veh + m), indicating that the change in genotype of D1R-cre mice had no effect on locomotor activity compared with WT mice (*P* > 0.05, Figures 2A, 3B,D and Supplementary Figures 3A,C). In terms of the results of the stereotyped behavior test, the score of stereotyped behavior in each IDPN-induced TS group [D1R-cre + IDPN + mCherry + i.p. Saline group (TS + m), D1R-cre + IDPN + Gq + i.p. Saline group (TS + Gq), and D1R-cre + IDPN + Gi + i.p. Saline group (TS + Gi)] was significantly higher than that of each vehicle group [D1R-cre + Vehicle + mCherry + i.p. Saline group (Veh + m), D1R-cre + Vehicle + Gq + i.p. Saline group (Veh + Gq), and D1R-cre + Vehicle + Gi + i.p. Saline group (Veh + Gi)] (*P* < 0.05, Figure 2A), indicating successful modeling.

Thirty minutes after the injection of CNO (i.p. CNO), the scores of stereotyped behavior in each IDPN-induced TS group (TS + m, TS + Gq, and TS + Gi) was significantly higher than those of each vehicle group (Veh + m, Veh + Gq, and Veh + Gi) (*P* < 0.05, Figure 2B), indicating that CNO did not affect the effect of IDPN on the behavioral stereotypies. There was no significant difference in the scores of stereotyped behavior between D1R-cre + IDPN + Gq + i.p. CNO (TS + Gq) and D1R-cre + IDPN + mCherry + i.p. CNO (TS + m) groups (*P* > 0.05, Figure 2B), indicating that chemogenetic activation of D1R-containing neurons in the SNpc did not affect the involuntary behavioral stereotypies in TS mice. Compared with the D1R-cre + IDPN + mCherry + i.p. CNO group (TS + m), the score of stereotyped behavior of D1R-cre + IDPN + Gi + i.p. CNO group (TS + Gi) was significantly decreased (*P* < 0.05, Figure 2B), indicating that chemogenetic inhibition of D1R-containing neurons in the SNpc significantly alleviated involuntary behavioral stereotypies in TS mice.

### Chemogenetic Activation of Dopaminergic D1 Receptor-Containing Neurons in the Dorsal Striatum Significantly Aggravated Involuntary Behavioral Stereotypies of Vehicle-Treated Mice, While Chemogenetic Inhibition of Dopaminergic D1 Receptor-Containing Neurons in the Dorsal Striatum Significantly Alleviated Involuntary Behavioral Stereotypies of Tourette Syndrome Mice

**FIGURE 4:**
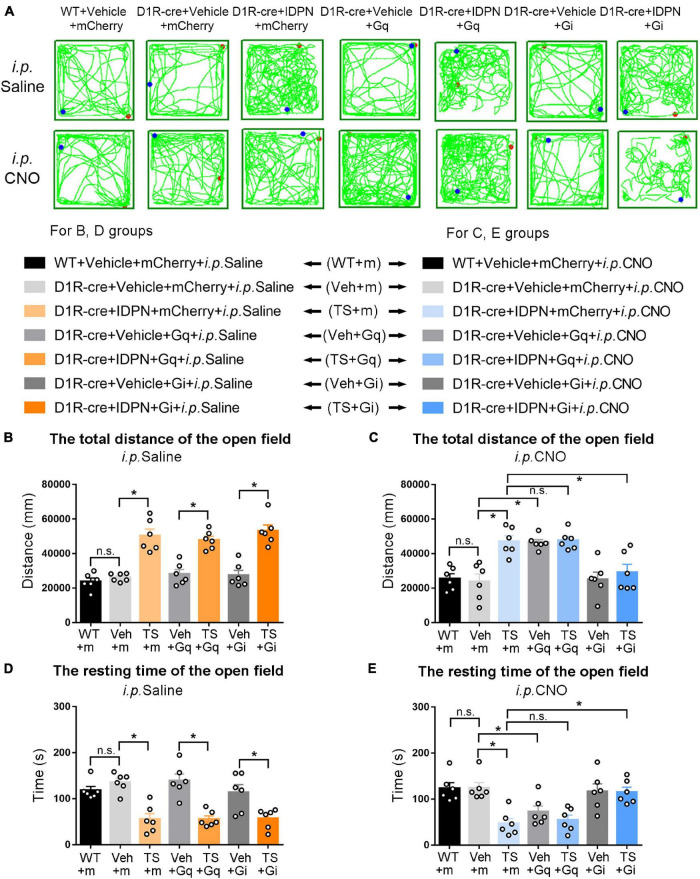
Effects of activation or inhibition of D1R-containing neurons in the dSTR on total distance and resting time in the open-field test in mice. **(A)** Evaluations of the trajectory in the open-field test in mice 30 min after the injection of saline (i.p. Saline) or CNO (i.p. CNO) in the WT + Vehicle + mCherry + i.p. Saline group (WT + m), D1R-cre + Vehicle + mCherry + i.p. Saline group (Veh + m), D1R-cre + IDPN + mCherry + i.p. Saline group (TS + m), D1R-cre + Vehicle + Gq + i.p. Saline group (Veh + Gq), D1R-cre + IDPN + Gq + i.p. Saline group (TS + Gq), D1R-cre + Vehicle + Gi + i.p. Saline group (Veh + Gi), D1R-cre + IDPN + Gi + i.p. Saline group (TS + Gi), WT + Vehicle + mCherry + i.p. CNO group (WT + m), D1R-cre + Vehicle + mCherry + i.p. CNO group (Veh + m), D1R-cre + IDPN + mCherry + i.p. CNO group (TS + m), D1R-cre + Vehicle + Gq + i.p. CNO group (Veh + Gq), D1R-cre + IDPN + Gq + i.p. CNO group (TS + Gq), D1R-cre + Vehicle + Gi + i.p. CNO group (Veh + Gi), D1R-cre + IDPN + Gi + i.p. CNO group (TS + Gi) on day 23. **(B)** Total distance in the open-field test within 10 min in mice of each group 30 min after the injection of saline (i.p. Saline) on day 23. **(C)** Total distance in the open-field test within 10 min in mice of each group 30 min after the injection of CNO (i.p. CNO) on day 23. **(D)** Resting time in the open-field test within 10 min in mice of each group 30 min after the injection of saline (i.p. Saline) on day 23. **(E)** Resting time in the open-field test within 10 min in mice of each group 30 min after the injection of CNO (i.p. CNO) on day 23. Data are expressed as mean ± SEM (*n* = 6 mice in each group), and the black line segment indicates the differences between groups, * represents *P* < 0.05 between marked groups, and n.s. represents *P* > 0.05 between marked groups.

The change in genotype of D1R-cre mice had no effect on locomotor activity compared with WT mice (*P* > 0.05, Figures 2C, 4B,D and Supplementary Figures 4A,C). In terms of the results of the stereotyped behavior test, after the injection of saline (i.p. Saline, contrast with CNO), the score of stereotyped behavior in each IDPN-induced TS model group was significantly higher than that of each vehicle group (*P* < 0.05, Figure 2C), indicating successful modeling.

After the injection of CNO (i.p. CNO), the score of stereotyped behavior of the D1R-cre + Vehicle + Gq + i.p. CNO group (Veh + Gq) was significantly higher than that of the D1R-cre + Vehicle + mCherry + i.p. CNO group (Veh + m) (*P* < 0.05, Figure 2D), indicating that chemogenetic activation of D1R-containing neurons in the dSTR significantly aggravated involuntary behavioral stereotypies in vehicle-treated mice. At the same time, there was no significant difference in the scores of stereotyped behavior between D1R-cre + Vehicle + Gi + i.p. CNO (Veh + Gi) and D1R-cre + Vehicle + mCherry + i.p. CNO (Veh + m) groups (*P* > 0.05, Figure 2D), indicating that chemogenetic inhibition of D1R-containing neurons in the dSTR had no significant effect on involuntary behavioral stereotypies in vehicle-treated mice.

Compared with the D1R-cre + IDPN + mCherry + i.p. CNO group (TS + m), the score of stereotyped behavior of the D1R-cre + IDPN + Gi + i.p. CNO group (TS + Gi) was significantly decreased (*P* < 0.05, Figure 2D), indicating that chemogenetic inhibition of D1R-containing neurons in the dSTR significantly alleviated the involuntary behavioral stereotypies in TS mice. There was no significant difference in the scores of stereotyped behavior between D1R-cre + IDPN + Gq + i.p. CNO (TS + Gq) and D1R-cre + IDPN + mCherry + i.p. CNO (TS + m) groups (*P* > 0.05, Figure 2D), indicating that chemogenetic activation of D1R-containing neurons in the dSTR did not affect the involuntary behavioral stereotypies in TS mice.

### Chemogenetic Inhibition of Dopaminergic D2 Receptor-Containing Neurons in the Substantia Nigra Pars Compacta Significantly Alleviated Involuntary Behavioral Stereotypies of Tourette Syndrome Mice

**FIGURE 5:**
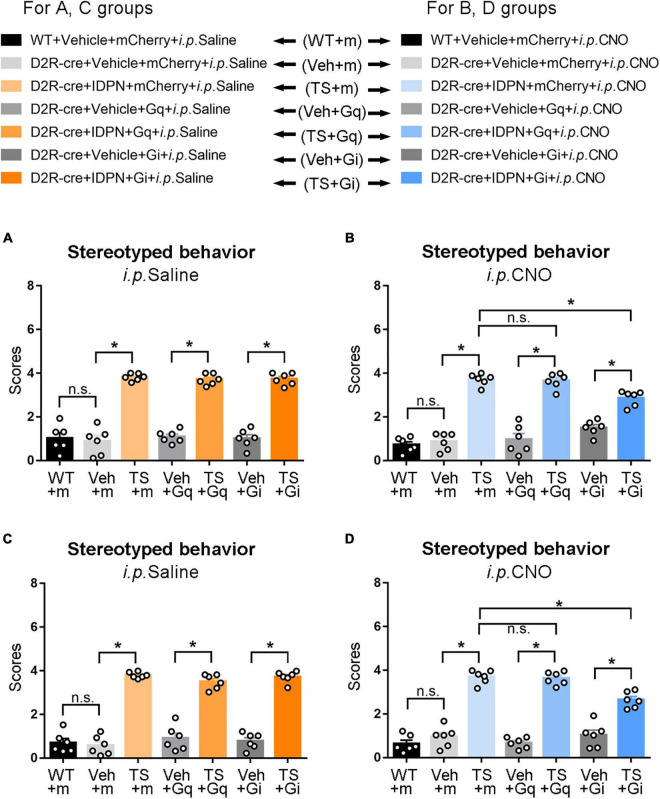
Effects of activation or inhibition of the D2R-containing neurons in the SNpc and dSTR on stereotyped behavior in mice. **(A)** Evaluations of stereotyped behavior scores of mice injected with virus in the SNpc 30 min after the injection of saline (i.p. Saline) in the WT + Vehicle + mCherry + i.p. Saline group (WT + m), D2R-cre + Vehicle + mCherry + i.p. Saline group (Veh + m), D2R-cre + IDPN + mCherry + i.p. Saline group (TS + m), D2R-cre + Vehicle + Gq + i.p. Saline group (Veh + Gq), D2R-cre + IDPN + Gq + i.p. Saline group (TS + Gq), D2R-cre + Vehicle + Gi + i.p. Saline group (Veh + Gi), D2R-cre + IDPN + Gi + i.p. Saline group (TS + Gi) on day 23. **(B)** Evaluations of stereotyped behavior scores of mice injected with virus in the SNpc 30 min after the injection of CNO (i.p. CNO) in the WT + Vehicle + mCherry + i.p. CNO group (WT + m), D2R-cre + Vehicle + mCherry + i.p. CNO group (Veh + m), D2R-cre + IDPN + mCherry + i.p. CNO group (TS + m), D2R-cre + Vehicle + Gq + i.p. CNO group (Veh + Gq), D2R-cre + IDPN + Gq + i.p. CNO group (TS + Gq), D2R-cre + Vehicle + Gi + i.p. CNO group (Veh + Gi), D2R-cre + IDPN + Gi + i.p. CNO group (TS + Gi) on day 23. **(C)** Evaluations of stereotyped behavior scores of mice injected with virus in the dSTR 30 min after the injection of saline (i.p. Saline) in each group on day 23. **(D)** Evaluations of stereotyped behavior scores of mice injected with virus in the dSTR 30 min after the injection of CNO (i.p. CNO) in each group on day 23. Data are expressed as mean ± SEM (*n* = 6 mice in each group), and the black line segment indicates the differences between groups, * represents *P* < 0.05 between marked groups, n.s. represents *P* > 0.05 between marked groups.

**FIGURE 6:**
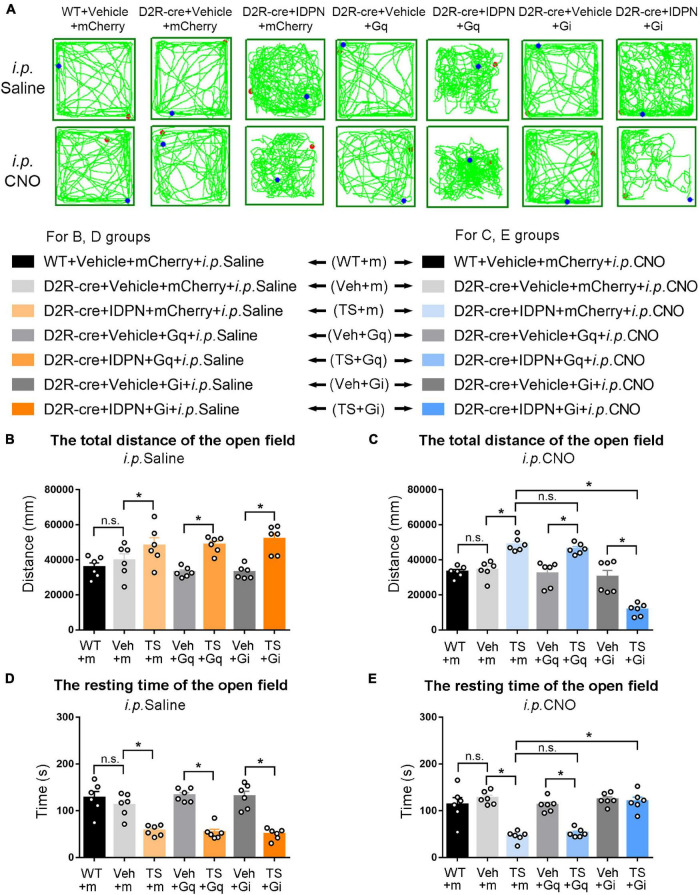
Effects of activation or inhibition of D2R-containing neurons in the SNpc on total distance and resting time in the open-field test in mice. **(A)** Evaluations of the trajectory in the open-field test in mice 30 min after the injection of saline (i.p. Saline) or CNO (i.p. CNO) in the WT + Vehicle + mCherry + i.p. Saline group (WT + m), D2R-cre + Vehicle + mCherry + i.p. Saline group (Veh + m), D2R-cre + IDPN + mCherry + i.p. Saline group (TS + m), D2R-cre + Vehicle + Gq + i.p. Saline group (Veh + Gq), D2R-cre + IDPN + Gq + i.p. Saline group (TS + Gq), D2R-cre + Vehicle + Gi + i.p. Saline group (Veh + Gi), D2R-cre + IDPN + Gi + i.p. Saline group (TS + Gi), WT + Vehicle + mCherry + i.p. CNO group (WT + m), D2R-cre + Vehicle + mCherry + i.p. CNO group (Veh + m), D2R-cre + IDPN + mCherry + i.p. CNO group (TS + m), D2R-cre + Vehicle + Gq + i.p. CNO group (Veh + Gq), D2R-cre + IDPN + Gq + i.p. CNO group (TS + Gq), D2R-cre + Vehicle + Gi + i.p. CNO group (Veh + Gi), D2R-cre + IDPN + Gi + i.p. CNO group (TS + Gi) on day 23. **(B)** Total distance in the open-field test within 10 min in mice of each group 30 min after the injection of saline (i.p. Saline) on day 23. **(C)** Total distance in the open-field test within 10 min in mice of each group 30 min after the injection of CNO (i.p. CNO) on day 23. **(D)** Resting time in the open-field test within 10 min in mice of each group 30 min after the injection of saline (i.p. Saline) on day 23. **(E)** Resting time in the open-field test within 10 min in mice of each group 30 min after the injection of CNO (i.p. CNO) on day 23. Data are expressed as mean ± SEM (*n* = 6 mice in each group), and the black line segment indicates the differences between groups, * represents *P* < 0.05 between marked groups, and n.s. represents *P* > 0.05 between marked groups.

The change in genotype of D2R-cre mice had no effect on locomotor activity compared with WT mice (*P* > 0.05, Figures 5A, 6B,D and Supplementary Figures 5A,C). The score of stereotyped behavior in each TS group was significantly higher than that of each vehicle group (*P* < 0.05, Figure 5A), indicating successful modeling.

After the injection of CNO (i.p. CNO), the score of stereotyped behavior in each IDPN-induced TS group (TS + m, TS + Gq, and TS + Gi) was significantly higher than that of each vehicle group (Veh + m, Veh + Gq, and Veh + Gi) (*P* < 0.05, Figure 5B), indicating that CNO did not affect the effect of IDPN on the TS model. Compared with the D2R-cre + IDPN + mCherry + i.p. CNO group (TS + m), the score of stereotyped behavior of D2R-cre + IDPN + Gi + i.p. CNO group (TS + Gi) was significantly decreased (*P* < 0.05, Figure 5B), indicating that chemogenetic inhibition of D2R-containing neurons in the SNpc significantly alleviated behavioral stereotypies in TS mice. There was no significant difference in the scores of stereotyped behavior between D2R-cre + IDPN + Gq + i.p. CNO (TS + Gq) and D2R-cre + IDPN + mCherry + i.p. CNO (TS + m) groups (*P* > 0.05, Figure 5B), indicating that chemogenetic activation of D2R-containing neurons in the SNpc did not affect the involuntary behavioral stereotypies in TS mice.

### Chemogenetic Inhibition of Dopaminergic D2 Receptor-Containing Neurons in the Dorsal Striatum Significantly Alleviated Involuntary Behavioral Stereotypies of Tourette Syndrome Mice

**FIGURE 7:**
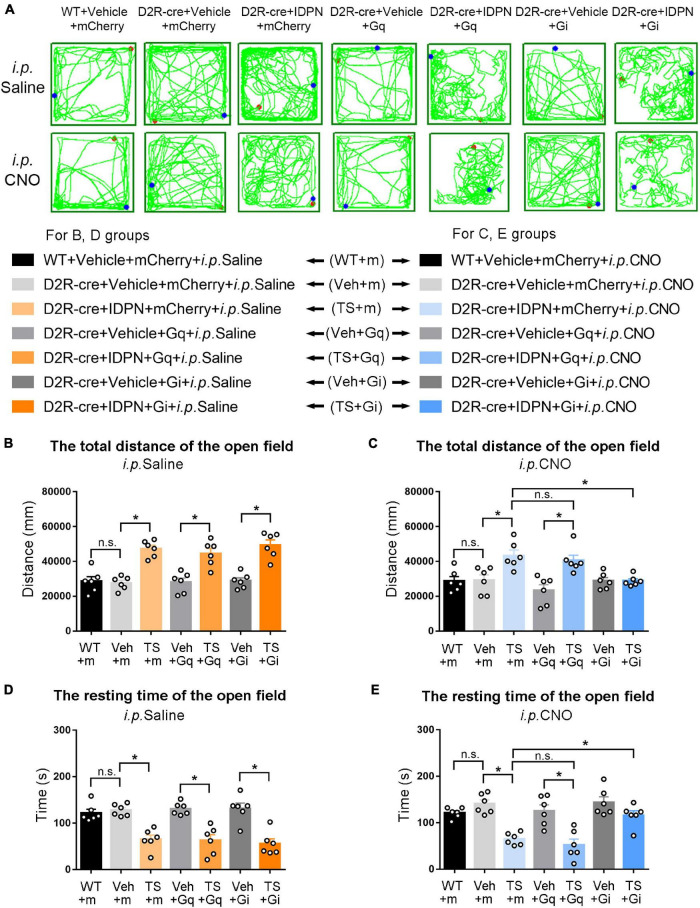
Effects of activation or inhibition of D2R-containing neurons in the dSTR on total distance and resting time in the open-field test in mice. **(A)** Evaluations of the trajectory in the open-field test in mice 30 min after the injection of saline (i.p. Saline) or CNO (i.p. CNO) in the WT + Vehicle + mCherry + i.p. Saline group (WT + m), D2R-cre + Vehicle + mCherry + i.p. Saline group (Veh + m), D2R-cre + IDPN + mCherry + i.p. Saline group (TS + m), D2R-cre + Vehicle + Gq + i.p. Saline group (Veh + Gq), D2R-cre + IDPN + Gq + i.p. Saline group (TS + Gq), D2R-cre + Vehicle + Gi + i.p. Saline group (Veh + Gi), D2R-cre + IDPN + Gi + i.p. Saline group (TS + Gi), WT + Vehicle + mCherry + i.p. CNO group (WT + m), D2R-cre + Vehicle + mCherry + i.p. CNO group (Veh + m), D2R-cre + IDPN + mCherry + i.p. CNO group (TS + m), D2R-cre + Vehicle + Gq + i.p. CNO group (Veh + Gq), D2R-cre + IDPN + Gq + i.p. CNO group (TS + Gq), D2R-cre + Vehicle + Gi + i.p. CNO group (Veh + Gi), D2R-cre + IDPN + Gi + i.p. CNO group (TS + Gi) on day 23. **(B)** Total distance in the open-field test within 10 min in mice of each group 30 min after the injection of saline (i.p. Saline) on day 23. **(C)** Total distance in the open-field test within 10 min in mice of each group 30 min after the injection of CNO (i.p. CNO) on day 23. **(D)** Resting time in the open-field test within 10 min in mice of each group 30 min after the injection of saline (i.p. Saline) on day 23. **(E)** Resting time in the open-field test within 10 min in mice of each group 30 min after the injection of CNO (i.p. CNO) on day 23. Data are expressed as mean ± SEM (*n* = 6 mice in each group), and the black line segment indicates the differences between groups, * represents *P* < 0.05 between marked groups, and n.s. represents *P* > 0.05 between marked groups.

The change in genotype of D2R-cre mice had no effect on locomotor activity compared with WT mice (*P* > 0.05, Figures 5C, 7B,D and Supplementary Figures 6A,C). After the injection of saline (i.p. Saline), the score of stereotyped behavior in each TS group was significantly higher than that of each vehicle group (*P* < 0.05, Figure 5C), indicating successful modeling.

After the injection of CNO (i.p. CNO), the score of stereotyped behavior in each IDPN-induced TS group (TS + m, TS + Gq, and TS + Gi) was significantly higher than that of each vehicle group (Veh + m, Veh + Gq, and Veh + Gi) (*P* < 0.05, Figure 5D), indicating that CNO did not affect the effect of IDPN on the TS model. Compared with the D2R-cre + IDPN + mCherry + i.p. CNO group (TS + m), the score of stereotyped behavior of D2R-cre + IDPN + Gi + i.p. CNO group (TS + Gi) was significantly decreased (*P* < 0.05, Figure 5D), indicating that chemogenetic inhibition of D2R-containing neurons in the dSTR significantly alleviated behavioral stereotypies in TS mice. There was no significant difference in the score of stereotyped behavior between D2R-cre + IDPN + Gq + i.p. CNO (TS + Gq) and D2R-cre + IDPN + mCherry + i.p. CNO (TS + m) groups (*P* > 0.05, Figure 5D), indicating that chemogenetic activation of D2R-containing neurons in the dSTR did not affect the involuntary behavioral stereotypies in TS mice.

### Chemogenetic Inhibition of Dopaminergic D1 Receptor- or Dopaminergic D2 Receptor-Containing Neurons in the Substantia Nigra Pars Compacta and Dorsal Striatum Decreased the Activity in the Open-Field Test of Tourette Syndrome Mice, While Chemogenetic Activation of Dopaminergic D1 Receptor-Containing Neurons in the Dorsal Striatum Increased the Activity in the Open-Field Test of Vehicle-Treated Mice

The results of total distance and resting time of mice in the open-field test were similar to those in the stereotyped behavior test (Figures 3, 4, 6, 7). In the open-field test (Figures 3, 4, 6, 7A was the trajectory in the open-field test), after the injection of saline (i.p. Saline), compared with each vehicle group (Veh + m, Veh + Gq and Veh + Gi), total distance in the open-field test within 10 min of each TS model group (TS + m, TS + Gq and TS + Gi) was significantly increased (*P* < 0.05, Figures 3, 4, 6, 7B), and resting time in the open-field test within 10 min was significantly decreased (*P* < 0.05, Figures 3, 4, 6, 7D), indicating that IDPN significantly increased the activity in mice.

After the injection of CNO (i.p. CNO), compared with the TS + m group, total distance of the TS + Gi group was significantly decreased (*P* < 0.05, Figures 3, 4, 6, 7C), and resting time was significantly increased (*P* < 0.05, Figures 3, 4, 6, 7E). It indicated that after inhibiting D1R- or D2R-containing neurons in the SNpc and dSTR, the activity in the open-field test in TS mice was decreased. Compared with the TS + m group, total distance and resting time of the TS + Gq group did not significantly change (*P* > 0.05, Figures 3, 6, 7C,E). It indicated that chemogenetic activation of D1R- or D2R-containing neurons in the SNpc- and D2R-containing neurons in the dSTR did not affect the activity in the open-field test in TS mice.

As shown in Figure 4, after the injection of CNO (i.p. CNO), compared with the D1R-cre + Vehicle + mCherry + i.p. CNO group (Veh + m), total distance of the D1R-cre + Vehicle + Gq + i.p. CNO group (Veh + Gq) was significantly increased (*P* < 0.05, Figure 4C), and resting time was significantly decreased (*P* < 0.05, Figure 4E) in the open-field test, indicating that after activating D1R-containing neurons in the dSTR, the activity in the open-field test of the vehicle-treated mice was increased. Compared with the D1R-cre + Vehicle + mCherry + i.p. CNO group (Veh + m), total distance and resting time in the open-field test of the D1R-cre + Vehicle + Gi + i.p. CNO group (Veh + Gi) did not significantly change (*P* > 0.05, Figures 4C,E). It indicated that chemogenetic inhibition of D1R-containing neurons in the dSTR did not affect the activity in the open-field test in vehicle-treated mice.

In addition, chemogenetic activation or inhibition of D1R- or D2R-containing neurons in the SNpc and dSTR had no significant effect on motor coordination (Supplementary Result Section “Chemogenetic Activation or Inhibition D1R or D2R-Containing Neurons in the SNpc and dSTR Had No Significant Effect on Motor Coordination and Grip Strength in Mice” and Supplementary Figures 3–6A,B) and grip strength (Supplementary Result Section “Chemogenetic Activation or Inhibition D1R or D2R-Containing Neurons in the SNpc and dSTR Had No Significant Effect on Motor Coordination and Grip Strength in Mice” and Supplementary Figures 3–6C,D) of mice.

## Discussion

Tourette syndrome is a common neurobehavioral disorder that is closely related to DA and its receptors (Nespoli et al., 2018). A large number of studies have found that dysfunction of the substantia nigra–striatum network could be the main pathogenesis of TS, which is closely associated with the hyperfunction of the nigrostriatal dopaminergic neurons (Zebardast et al., 2013). Our study showed that chemogenetic inhibition of D1R- or D2R-containing neurons in the SNpc and dSTR significantly alleviated involuntary behavioral stereotypies in TS mice. Chemogenetically activating D1R-containing neurons, but not D2R-containing neurons, in the dSTR significantly aggravated behavioral stereotypies in vehicle-treated mice. Chemogenetic activation or inhibition of D1R- or D2R-containing neurons in the SNpc and dSTR had no significant effect on the motor coordination and grip strength of mice, and the results of total distance and the resting time of mice in the open-field test were similar to those of the stereotyped behavior test (Supplementary Table 1).

Previous studies have shown that the basal ganglia can control different processes of movement by regulating the DA system, thus playing an important role in the pathophysiology of TS (Buot and Yelnik, 2012). The main pathogenesis of TS is the hyperfunction of dopaminergic neurons projecting from the substantia nigra to the striatum (Cui Y. H. et al., 2013), leading to an increased release of DA in the substantia nigra–striatum network, thereby causing TS (Maqsood et al., 2020).

**FIGURE 8:**
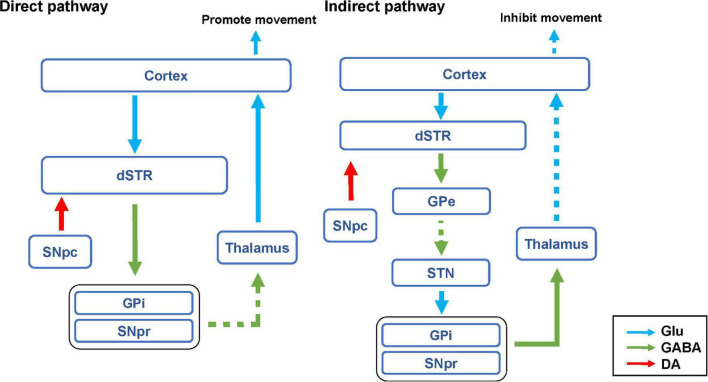
Hypothesis diagram of direct and indirect pathways of basal ganglia motor regulatory system. The blue arrow represents glutamatergic (Glu) neuron transmission. The green arrow indicates GABAergic neuron transmission. The red arrow represents dopaminergic neuron transmission. The solid line represents activation. The dashed line indicates inhibition.

The basal ganglia motor system includes both direct and indirect pathways. D1R and D2R are expressed in the direct and indirect pathways, respectively (Beaulieu and Gainetdinov, 2011). In the direct pathway, the SNpc releases a large amount of DA to activate GABAergic projection neurons in the striatum, which can inhibit the globus pallidus internus (GPi) and substantia nigra pars reticulata (SNpr) (Grillner and Robertson, 2016). The thalamus is normally inhibited by the globus pallidus and the SNpr. When the direct pathway is activated, the thalamus is disinhibited and it sends excitatory impulses back to the cortex, thus promoting movement (Figure 8; Cui G. et al., 2013; Dobbs et al., 2016).

**FIGURE 9:**
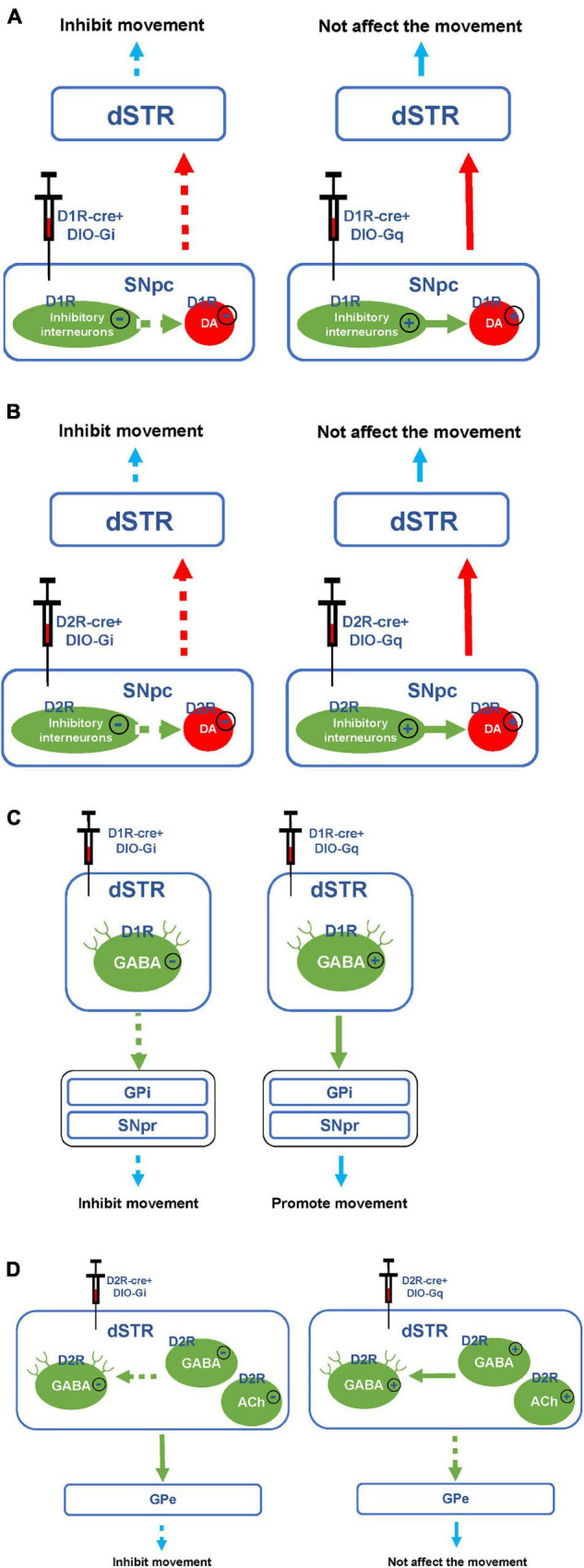
Hypothesis diagram of experimental mechanism. **(A)** Hypothesis diagram of chemogenetic inhibition or activation of D1R-containing neurons in the SNpc. **(B)** Hypothesis diagram of chemogenetic inhibition or activation of D2R-containing neurons in the SNpc. **(C)** Hypothesis diagram of chemogenetic inhibition or activation of D1R-containing neurons in the dSTR. **(D)** Hypothesis diagram of chemogenetic inhibition or activation of D2R-containing neurons in the dSTR. The solid line or plus sign indicates activation. The dashed line or minus sign indicates inhibition.

In this study, we found that chemogenetic inhibition of D1R- or D2R-containing neurons in the SNpc significantly alleviated behavioral stereotypies in TS mice. In the SNpc, D1R, and D2R are expressed on both dopaminergic neurons and inhibitory interneurons (Figures 9A,B; Yung et al., 1995; Yang et al., 2019). Chemogenetic inhibition of D1R- or D2R-containing dopaminergic neurons may inhibit the release of DA in the direct pathway and reduce movement, thus alleviating involuntary behavioral stereotypies. Although chemogenetic inhibition of D1R- or D2R-containing inhibitory interneurons may disinhibit D1R or D2R-containing dopaminergic neurons, dopaminergic neurons were already inhibited by chemogenetic approach and could not be activated. In our study, D1R- or D2R-containing neurons were manipulating, not D1R or D2R receptors. As a result, chemogenetic inhibition of D1R- or D2R-containing neurons in the SNpc produced the same effects of reducing movement. However, chemogenetic activation of D1R- or D2R-containing neurons in the SNpc did not aggravate the behavioral stereotypies of vehicle-treated and TS mice. It has been demonstrated that D1R or D2R is also expressed in inhibitory interneurons in the SNpc (Cover et al., 2019; Ohira, 2019) and can be activated by a chemogenetic approach to inhibit D1R- or D2R-containing dopaminergic neurons (Figures 9A,B). Therefore, chemogenetic activation of D1R- or D2R-containing neurons did not affect movement.

In our study, after inhibiting or activating D1R-containing neurons in the dSTR, the stereotyped behavior of TS or vehicle-treated mice was significantly decreased or increased, respectively. Since D1R is mainly expressed on GABAergic projection neurons but not inhibitory interneurons in the dSTR (Figure 9C; Avila-Luna et al., 2019; Assali et al., 2021), chemogenetic inhibition of D1R-containing neurons in the dSTR may inhibit the direct pathway, thus alleviating the stereotyped behavior of the TS model. Although chemogenetically activating D1R-containing neurons might have enhance the direct pathway, it only induced stereotyped behavior in vehicle-treated mice, but did not deteriorate the stereotyped behavior of TS mice. This might be the reason why the behavioral stereotypies of TS mice reached a peak and would not increase further by chemogenetic activation. This suggests that the activation of D1R-containing neurons in the dSTR may be involved in the pathogenesis of TS.

As shown in Figure 8, in the indirect pathway, the striatum transmits inhibitory efferent stimuli to the globus pallidus externus (GPe), which in turn disinhibits the subthalamic nucleus (STN), leading to increased excitement of the GPi and SNpr, thus inhibiting the thalamus and weakening the excitement of the cortex (Hadipour-Niktarash et al., 2012). Therefore, the activation of the indirect pathway can inhibit the movement (Freeze et al., 2013). In this study, we found that chemogenetic activation of D2R-containing neurons in the dSTR did not significantly affect the behavioral stereotypies of vehicle-treated mice. This may be due to the activation of various types of D2R-containing neurons in the dSTR, including GABAergic projection neurons that can inhibit movement in indirect pathways, as well as inhibitory GABAergic or acetylcholinergic interneurons, which can inhibit GABAergic projection neurons (Figure 9D; Alcantara et al., 2003; Maurice et al., 2004; Zhang et al., 2009; Jijon-Lorenzo et al., 2018; Brandenburg et al., 2020; Lewis et al., 2020). Since D2R-containing projection and interneurons played the opposite role in movement, chemogenetic activation of D2R-containing neurons in the dSTR had no significant effect on behavioral stereotypies in mice.

Previous studies have found that enhanced GABA transmission drives bradykinesia following the loss of D2R signaling in medium spiny neurons of the indirect pathway (Lemos et al., 2016). After inhibiting D2R-containing neurons in the dSTR, the involuntary behavioral stereotypies of TS mice were alleviated. This result was different from what we expected, we speculated that this may be because the number of D2R-containing inhibitory interneurons in the dSTR exceeds that of GABAergic projection neurons. After both are chemogenetically inhibited, the activity of inhibitory interneurons is greatly reduced, and GABAergic projection neurons in the indirect pathway are disinhibited, thereby inhibiting movement (Figure 9D). Since we found that chemogenetic inhibition of D1R- or D2R-containing neurons in the SNpc and dSTR can alleviate involuntary behavioral stereotypies in TS mice, we speculated that inhibiting D1R- and D2R-containing neurons in the SNpc and dSTR at the same time may have a better therapeutic effect.

In this study, after inhibiting D1R- or D2R-containing neurons in the SNpc or dSTR, the total distance of TS mice in the open-field test was decreased and the resting time was increased, indicating that spontaneous locomotor activities of TS mice were reduced. After activating D1R- but not D2R-containing neurons in the dSTR, the spontaneous locomotor activities of vehicle-treated mice in the open-field test were increased, which was similar to the result of the stereotyped behavior test and mainly due to the expression of D2R but not D1R on inhibitory interneurons in the dSTR. The rotarod test was used to evaluate the motor coordination in mice. Previous studies have shown that chemogenetic activation or inhibition of direct or indirect pathways does not impair motor coordination (Kravitz et al., 2010). Our study found that after activating or inhibiting D1R- or D2R-containing neurons in the SNpc or dSTR, the movement coordination of mice in each group was not affected, which was consistent with previous studies.

Previous studies have demonstrated that D1R and D2R antagonists have extrapyramidal side effects (Peacock et al., 1999). The clinical use of dopaminergic agonists or antagonists can disturb the regulation of blood pressure leading to either hypo- or hypertension, respectively (Gonsai et al., 2018). In addition, chemogenetic strategy is a more precise intervention on a specific target, which is the advantages of this technology. Therefore, we used chemogenetic strategies to selectively activate or inhibit D1R- or D2R-containing neurons in the SNpc or dSTR, which also provided guidance on the clinical treatment of TS, such as deep brain stimulation (DBS). The targets applied for DBS in TS include the STN, the GPi, and the GPe (Rotsides and Mammis, 2013; Andrade and Visser-Vandewalle, 2016; Zhu et al., 2019); the exploration of these brain regions will become our next research direction.

In conclusion, we found that chemogenetic inhibition of D1R- or D2R-containing neurons in the SNpc and dSTR significantly alleviated involuntary behavioral stereotypies in TS mice, which may provide a new approach for the treatment of TS, and help overcome the clinical problem of TS. Moreover, the activation of D1R-containing neurons in the dSTR could also contribute to the occurrence of TS, which may be a more specific target for TS treatment.

## Data Availability Statement

The original contributions presented in the study are included in the article/Supplementary Material, further inquiries can be directed to the corresponding authors.

## Ethics Statement

All animal experimental protocols conformed to the Animal Management Rules of the Chinese Ministry of Health, and the study was approved by the Animal Ethics Committee of the Chinese Academy of Medical Sciences.

## Author Contributions

ML and WS conceived and designed the experiments. LL did most of the experiments and analyzed the data. YL, SW, and WW helped with the behavior test experiments. HeZ, LY, and YS helped with the viruses constructs and surgery experiments. HX and TH helped with the immunofluorescence labeling experiments. HoZ and YM helped with analyzing the data. LL, ML, and WS wrote the manuscript. All authors reviewed the manuscript.

## Conflict of Interest

The authors declare that the research was conducted in the absence of any commercial or financial relationships that could be construed as a potential conflict of interest.

## Publisher’s Note

All claims expressed in this article are solely those of the authors and do not necessarily represent those of their affiliated organizations, or those of the publisher, the editors and the reviewers. Any product that may be evaluated in this article, or claim that may be made by its manufacturer, is not guaranteed or endorsed by the publisher.

## References

[B1] Alcantara A. A. Chen V. Herring B. E. Mendenhall J. M. Berlanga M. L. (2003). Localization of dopamine D2 receptors on cholinergic interneurons of the dorsal striatum and nucleus accumbens of the rat. *Brain Res.* 986 22–29. 10.1016/s0006-8993(03)03165-2 12965226

[B2] Andrade P. Visser-Vandewalle V. (2016). DBS in tourette syndrome: where are we standing now? *J. Neural Transm.* 123 791–796. 10.1007/s00702-016-1569-7 27209036

[B3] Assali D. R. Sidikpramana M. Villa A. P. Falkenstein J. Steele A. D. (2021). Type 1 dopamine receptor (D1R)-independent circadian food anticipatory activity in mice. *PLoS One* 16:e0242897. 10.1371/journal.pone.0242897 33556069 PMC7869994

[B4] Avila-Luna A. Rios C. Galvez-Rosas A. Montes S. Arias-Montano J. A. Bueno-Nava A. (2019). Chronic administration of the histamine H3 receptor agonist immepip decreases L-Dopa-induced dyskinesias in 6-hydroxydopamine-lesioned rats. *Psychopharmacology* 236 1937–1948. 10.1007/s00213-019-5182-y 30762089

[B5] Baym C. L. Corbett B. A. Wright S. B. Bunge S. A. (2008). Neural correlates of tic severity and cognitive control in children with Tourette syndrome. *Brain* 131(Pt 1), 165–179. 10.1093/brain/awm278 18056159

[B6] Beaulieu J. M. Gainetdinov R. R. (2011). The physiology, signaling, and pharmacology of dopamine receptors. *Pharmacol. Rev.* 63 182–217. 10.1124/pr.110.002642 21303898

[B7] Brandenburg C. Soghomonian J. J. Zhang K. Sulkaj I. Randolph B. Kachadoorian M. (2020). Increased Dopamine Type 2 gene expression in the dorsal striatum in individuals with autism spectrum disorder suggests alterations in indirect pathway signaling and circuitry. *Front. Cell Neurosci.* 14:577858. 10.3389/fncel.2020.577858 33240045 PMC7681004

[B8] Broderick P. A. Phelix C. F. (1997). I. Serotonin (5-HT) within dopamine reward circuits signals open-field behavior. II. Basis for 5-HT–DA interaction in cocaine dysfunctional behavior. *Neurosci. Biobehav. Rev.* 21 227–260. 10.1016/s0149-7634(96)00048-6 9168262

[B9] Bromberg-Martin E. S. Matsumoto M. Hikosaka O. (2010). Dopamine in motivational control: rewarding, aversive, and alerting. *Neuron* 68 815–834. 10.1016/j.neuron.2010.11.022 21144997 PMC3032992

[B10] Buot A. Yelnik J. (2012). Functional anatomy of the basal ganglia: limbic aspects. *Rev. Neurol.* 168 569–575. 10.1016/j.neurol.2012.06.015 22902172

[B11] Calabrese V. Di Maio A. Marino G. Cardinale A. Natale G. De Rosa A. (2020). Rapamycin, by Inhibiting mTORC1 signaling, prevents the loss of striatal bidirectional synaptic plasticity in a rat model of L-DOPA-Induced Dyskinesia. *Front. Aging Neurosci.* 12:230. 10.3389/fnagi.2020.00230 32848709 PMC7431470

[B12] Cen S. S. Yu J. Wang Q. Deeb W. Wang K. L. Shukla A. W. (2020). Multidisciplinary telemedicine care for tourette syndrome: minireview. *Front. Neurol.* 11:573576. 10.3389/fneur.2020.573576 33391146 PMC7775481

[B13] Chen J. Leong P. K. Leung H. Y. Chan W. M. Li Z. Qiu J. (2019). A chinese herbal formulation, xiao-er-an-shen decoction, attenuates tourette syndrome, possibly by reversing abnormal changes in neurotransmitter levels and enhancing antioxidant status in mouse brain. *Front. Pharmacol.* 10:812. 10.3389/fphar.2019.00812 31396086 PMC6667554

[B14] Cover K. K. Gyawali U. Kerkhoff W. G. Patton M. H. Mu C. White M. G. (2019). Activation of the rostral intralaminar thalamus drives reinforcement through striatal dopamine release. *Cell Rep.* 26 1389–1398 e1383. 10.1016/j.celrep.2019.01.044 30726725 PMC6402336

[B15] Cui G. Jun S. B. Jin X. Pham M. D. Vogel S. S. Lovinger D. M. (2013). Concurrent activation of striatal direct and indirect pathways during action initiation. *Nature* 494 238–242. 10.1038/nature11846 23354054 PMC4039389

[B16] Cui Y. H. Zheng Y. Jin Z. He Y. Chen X. Yu L. P. (2013). [Relationship between tic symptom severity and amplitude of low frequency fluctuation of resting-state functional magnetic resonance imaging of Tourette syndrome]. *Zhonghua Er Ke Za Zhi* 51 448–452. 24120064

[B17] Dobbs L. K. Kaplan A. R. Lemos J. C. Matsui A. Rubinstein M. Alvarez V. A. (2016). Dopamine regulation of lateral inhibition between striatal neurons gates the stimulant actions of cocaine. *Neuron* 90 1100–1113. 10.1016/j.neuron.2016.04.031 27181061 PMC4891261

[B18] Dodel I. Reese J. P. Muller N. Munchau A. Balzer-Geldsetzer M. Wasem J. (2010). Cost of illness in patients with Gilles de la Tourette’s syndrome. *J. Neurol.* 257 1055–1061. 20179960 10.1007/s00415-010-5458-y

[B19] Egolf A. Coffey B. J. (2014). Current pharmacotherapeutic approaches for the treatment of Tourette syndrome. *Drugs Today* 50 159–179. 10.1358/dot.2014.50.2.2097801 24619591

[B20] Ferguson S. M. Eskenazi D. Ishikawa M. Wanat M. J. Phillips P. E. Dong Y. (2011). Transient neuronal inhibition reveals opposing roles of indirect and direct pathways in sensitization. *Nat. Neurosci.* 14 22–24. 10.1038/nn.2703 21131952 PMC3058296

[B21] Freeze B. S. Kravitz A. V. Hammack N. Berke J. D. Kreitzer A. C. (2013). Control of basal ganglia output by direct and indirect pathway projection neurons. *J. Neurosci.* 33 18531–18539. 10.1523/jneurosci.1278-13.2013 24259575 PMC3834057

[B22] Ganos C. Martino D. (2015). Tics and tourette syndrome. *Neurol. Clin.* 33 115–136. 25432726 10.1016/j.ncl.2014.09.008

[B23] Gonsai N. H. Amin V. H. Mendpara C. G. Speth R. Hale G. M. (2018). Effects of dopamine receptor antagonist antipsychotic therapy on blood pressure. *J. Clin. Pharm. Ther.* 43 1–7. 10.1111/jcpt.12649 29119585

[B24] Grillner S. Robertson B. (2016). The basal ganglia over 500 Million years. *Curr. Biol.* 26 R1088–R1100. 10.1016/j.baga.2011.01.090 27780050

[B25] Groenewegen H. J. van den Heuvel O. A. Cath D. C. Voorn P. Veltman D. J. (2003). Does an imbalance between the dorsal and ventral striatopallidal systems play a role in Tourette’s syndrome? a neuronal circuit approach. *Brain Dev.* 25(Suppl. 1), S3–S14. 10.1016/s0387-7604(03)90001-5 14980365

[B26] Hadipour-Niktarash A. Rommelfanger K. S. Masilamoni G. J. Smith Y. Wichmann T. (2012). Extrastriatal D2-like receptors modulate basal ganglia pathways in normal and Parkinsonian monkeys. *J. Neurophysiol.* 107 1500–1512. 10.1152/jn.00348.2011 22131382 PMC3311684

[B27] Hamamoto Y. Kano Y. (2018). [Tourette Syndrome]. *Brain Nerve* 70 1237–1245. 30416117 10.11477/mf.1416201169

[B28] Hsu C. J. Wong L. C. Lee W. T. (2021). Immunological dysfunction in tourette syndrome and related disorders. *Int. J. Mol. Sci.* 22:853. 10.3390/ijms22020853 33467014 PMC7839977

[B29] Jakubovski E. Muller-Vahl K. R. (2017). [Gilles de la Tourette syndrome: symptoms, causes and therapy]. *Psychother. Psychosom. Med. Psychol.* 67 252–268. 28722101 10.1055/s-0043-103269

[B30] Jijon-Lorenzo R. Caballero-Floran I. H. Recillas-Morales S. Cortes H. Avalos-Fuentes J. A. Paz-Bermudez F. J. (2018). Presynaptic Dopamine D2 receptors modulate [(3)H]GABA release at striatopallidal terminals via activation of PLC–>IP3–>calcineurin and inhibition of AC–>cAMP–>PKA signaling cascades. *Neuroscience* 372 74–86. 10.1016/j.neuroscience.2017.12.041 29292080

[B31] Kawohl W. Bruhl A. Krowatschek G. Ketteler D. Herwig U. (2009). Functional magnetic resonance imaging of tics and tic suppression in Gilles de la Tourette syndrome. *World J. Biol. Psychiatry* 10(4 Pt 2), 567–570. 10.1080/15622970802118356 18609432

[B32] Khan H. A. Ibrahim K. E. (2015). Pattern of neurobehavioral and organ-specific toxicities of beta, beta’-iminodipropionitrile in mice. *Arch Med. Sci.* 11 1137–1144. 10.5114/aoms.2015.54871 26528360 PMC4624758

[B33] Kravitz A. V. Freeze B. S. Parker P. R. Kay K. Thwin M. T. Deisseroth K. (2010). Regulation of parkinsonian motor behaviours by optogenetic control of basal ganglia circuitry. *Nature* 466 622–626. 10.1038/nature09159 20613723 PMC3552484

[B34] Lemos J. C. Friend D. M. Kaplan A. R. Shin J. H. Rubinstein M. Kravitz A. V. (2016). Enhanced GABA transmission drives bradykinesia following loss of dopamine D2 receptor signaling. *Neuron* 90 824–838. 10.1016/j.neuron.2016.04.040 27196975 PMC4882167

[B35] Lerner A. Bagic A. Simmons J. M. Mari Z. Bonne O. Xu B. (2012). Widespread abnormality of the gamma-aminobutyric acid-ergic system in Tourette syndrome. *Brain* 135(Pt 6), 1926–1936. 10.1093/brain/aws104 22577221 PMC3359755

[B36] Lewis R. G. Serra M. Radl D. Gori M. Tran C. Michalak S. E. (2020). Dopaminergic control of striatal cholinergic interneurons underlies cocaine-induced psychostimulation. *Cell Rep.* 31:107527. 10.1016/j.celrep.2020.107527 32320647

[B37] Li Z. Chen Z. Fan G. Li A. Yuan J. Xu T. (2018). Cell-type-specific afferent innervation of the nucleus accumbens core and shell. *Front. Neuroanat.* 12:84. 10.3389/fnana.2018.00084 30459564 PMC6232828

[B38] Lin L. Yu L. Xiang H. Hu X. Yuan X. Zhu H. (2019). Effects of acupuncture on behavioral stereotypies and brain dopamine system in mice as a model of tourette syndrome. *Front. Behav. Neurosci.* 13:239. 10.3389/fnbeh.2019.00239 31680895 PMC6803462

[B39] Liu S. Tian M. He F. Li J. Xie H. Liu W. (2020). Mutations in ASH1L confer susceptibility to Tourette syndrome. *Mol. Psychiatry* 25 476–490. 10.1038/s41380-019-0560-8 31673123

[B40] Liu X. Wang X. Cao A. Zhang X. (2021). Immune function changes of the IDPN-induced Tourette syndrome rat model. *Int. J. Dev. Neurosci.* 81 159–166. 10.1002/jdn.10085 33377196

[B41] Lobo M. K. Covington H. E. III Chaudhury D. Friedman A. K. Sun H. Damez-Werno D. (2010). Cell type-specific loss of BDNF signaling mimics optogenetic control of cocaine reward. *Science* 330 385–390. 10.1126/science.1188472 20947769 PMC3011229

[B42] Maqsood A. Akram S. Akram F. (2020). Chlorpromazine-induced relapse of tourette syndrome in a patient with intellectual disability, attention deficit hyperactivity disorder, and Schizophrenia. *Cureus* 12:e10732. 10.7759/cureus.10732 33145137 PMC7599051

[B43] Maurice N. Mercer J. Chan C. S. Hernandez-Lopez S. Held J. Tkatch T. (2004). D2 dopamine receptor-mediated modulation of voltage-dependent Na+ channels reduces autonomous activity in striatal cholinergic interneurons. *J. Neurosci.* 24 10289–10301. 10.1523/JNEUROSCI.2155-04.2004 15548642 PMC6730305

[B44] Mogwitz S. Buse J. Ehrlich S. Roessner V. (2013). Clinical pharmacology of dopamine-modulating agents in Tourette’s syndrome. *Int. Rev. Neurobiol.* 112 281–349. 10.1016/B978-0-12-411546-0.00010-X 24295625

[B45] Nespoli E. Rizzo F. Boeckers T. Schulze U. Hengerer B. (2018). Altered dopaminergic regulation of the dorsal striatum is able to induce tic-like movements in juvenile rats. *PLoS One* 13:e0196515. 10.1371/journal.pone.0196515 29698507 PMC5919623

[B46] Ohira K. (2019). Dopamine stimulates differentiation and migration of cortical interneurons. *Biochem. Biophys. Res. Commun.* 512 577–583. 10.1016/j.bbrc.2019.03.105 30910356

[B47] Peacock L. Jensen G. Nicholson K. Gerlach J. (1999). Extrapyramidal side effects during chronic combined dopamine D1 and D2 antagonist treatment in *Cebus apella* monkeys. *Eur. Arch. Psychiatry Clin. Neurosci.* 249 221–226. 10.1007/s004060050090 10591986

[B48] Pogorelov V. Xu M. Smith H. R. Buchanan G. F. Pittenger C. (2015). Corticostriatal interactions in the generation of tic-like behaviors after local striatal disinhibition. *Exp. Neurol.* 265 122–128. 10.1016/j.expneurol.2015.01.001 25597650 PMC4361636

[B49] Proietti Onori M. Ceci C. Laviola G. Macri S. (2014). A behavioural test battery to investigate tic-like symptoms, stereotypies, attentional capabilities, and spontaneous locomotion in different mouse strains. *Behav. Brain Res.* 267 95–105. 10.1016/j.bbr.2014.03.023 24675156

[B50] Rodrigues S. Salum C. Ferreira T. L. (2017). Dorsal striatum D1-expressing neurons are involved with sensorimotor gating on prepulse inhibition test. *J. Psychopharmacol.* 31 505–513. 10.1177/0269881116686879 28114835

[B51] Rotsides J. Mammis A. (2013). The use of deep brain stimulation in Tourette’s syndrome. *Neurosurg. Focus* 35:E4. 10.3171/2013.8.FOCUS13292 24175864

[B52] Silva R. R. Munoz D. M. Daniel W. Barickman J. Friedhoff A. J. (1996). Causes of haloperidol discontinuation in patients with Tourette’s disorder: management and alternatives. *J. Clin. Psychiatry* 57 129–135. 8617698

[B53] Surmeier D. J. Ding J. Day M. Wang Z. Shen W. (2007). D1 and D2 dopamine-receptor modulation of striatal glutamatergic signaling in striatal medium spiny neurons. *Trends Neurosci.* 30 228–235. 17408758 10.1016/j.tins.2007.03.008

[B54] Wang D. H. Li W. Liu X. F. Zhang J. M. Wang S. M. (2013). Chinese medicine formula “Jian-Pi-Zhi-Dong Decoction” attenuates tourette syndrome via downregulating the expression of dopamine transporter in mice. *Evid. Based Complement. Alternat. Med.* 2013:385685. 10.1155/2013/385685 23431337 PMC3574653

[B55] Wang Y. Zhao L. Li A. Y. (2021). Gastrodin - A potential drug used for the treatment of Tourette Syndrome. *J. Pharmacol. Sci.* 145 289–295. 10.1016/j.jphs.2021.01.005 33602510

[B56] Xie H. Wang Z. Ji Y. Yin J. Yang W. H. Ren L. M. (2016). [Effects of salidroside on tic behavior of tourette syndrome model rats]. *Zhongguo Zhong Xi Yi Jie He Za Zhi* 36 90–93. 26955685

[B57] Yang C. Zhang L. Zhu P. Zhu C. Guo Q. (2016). The prevalence of tic disorders for children in China: a systematic review and meta-analysis. *Medicine* 95:e4354. 10.1097/MD.0000000000004354 27472724 PMC5265861

[B58] Yang W. Munhall A. C. Johnson S. W. (2019). AMP-activated protein kinase slows D2 dopamine autoreceptor desensitization in substantia nigra neurons. *Neuropharmacology* 158:107705. 10.1016/j.neuropharm.2019.107705 31301335 PMC6745265

[B59] Yung K. K. Bolam J. P. Smith A. D. Hersch S. M. Ciliax B. J. Levey A. I. (1995). Immunocytochemical localization of D1 and D2 dopamine receptors in the basal ganglia of the rat: light and electron microscopy. *Neuroscience* 65 709–730. 10.1016/0306-4522(94)00536-e 7609871

[B60] Zebardast N. Crowley M. J. Bloch M. H. Mayes L. C. Wyk B. V. Leckman J. F. (2013). Brain mechanisms for prepulse inhibition in adults with Tourette syndrome: initial findings. *Psychiatry Res.* 214 33–41. 10.1016/j.pscychresns.2013.05.009 23916249 PMC3932431

[B61] Zhang F. Li A. (2015a). Dual ameliorative effects of Ningdong granule on dopamine in rat models of Tourette’s syndrome. *Sci. Rep.* 5:7731. 10.1038/srep07731 25592875 PMC4296291

[B62] Zhang F. Li A. (2015b). Dual regulating effects of gastrodin on extracellular dopamine concentration in rats models of Tourette’s syndrome. *Int. J. Neurosci.* 125 784–792. 10.3109/00207454.2014.971455 25271797

[B63] Zhang F. Li A. (2015c). Dual restoring effects of gastrodin on dopamine in rat models of Tourette’s syndrome. *Neurosci. Lett.* 588 62–66. 10.1016/j.neulet.2014.12.051 25549540

[B64] Zhang T. Zhang L. Liang Y. Siapas A. G. Zhou F. M. Dani J. A. (2009). Dopamine signaling differences in the nucleus accumbens and dorsal striatum exploited by nicotine. *J. Neurosci.* 29 4035–4043. 10.1523/JNEUROSCI.0261-09.2009 19339599 PMC2743099

[B65] Zhang W. Yu W. Wang D. Wei L. Lee M. Wang S. (2014). Effect of “jian-pi-zhi-dong decoction” on gamma-aminobutyric Acid in a mouse model of Tourette syndrome. *Evid. Based Complement. Alternat. Med.* 2014:407509. 10.1155/2014/407509 24812567 PMC4000640

[B66] Zhao L. Cheng N. Sun B. Wang S. Li A. Wang Z. (2020). Regulatory effects of Ningdong granule on microglia-mediated neuroinflammation in a rat model of Tourette’s syndrome. *Biosci. Trends* 14 271–278. 10.5582/bst.2020.03262 32741856

[B67] Zhu G. Y. Geng X. Y. Zhang R. L. Chen Y. C. Liu Y. Y. Wang S. Y. (2019). Deep brain stimulation modulates pallidal and subthalamic neural oscillations in Tourette’s syndrome. *Brain Behav.* 9:e01450. 10.1002/brb3.1450 PMC6908859 31647199

