# Supplementary material for: Effects of Chemogenetic Inhibition of D1 or D2 Receptor-Containing Neurons of the Substantia Nigra and Striatum in Mice With Tourette Syndrome
Source: Front Mol Neurosci. 2021 Dec 9;14:779436. doi: 10.3389/fnmol.2021.779436 (PMC8696039; doi:10.3389/fnmol.2021.779436)
Supplement: Supplementary file 1 [file Data_Sheet_1.docx]

Supplementary Material

# Supplementary Results

## Chemogenetic activation or inhibition D1R or D2R-containing neurons in the SNpc and dSTR had no significant effect on motor coordination and grip strength in mice.

After injection of saline (i.p. Saline), compared with each vehicle group (Veh+m, Veh+Gq and Veh+Gi), the retention time on the rotarod in each IDPN-induced TS model group (TS+m, TS+Gq and TS+Gi) was significantly reduced (P < 0.05, **Supplementary** **Figures 3-6A**), indicating that motor coordination in TS mice was significantly weakened after IDPN modeling.

After injection of CNO (i.p. CNO), the retention time on the rotarod in each IDPN-induced TS model group (TS+m, TS+Gq and TS+Gi) was significantly shortened than that of each vehicle group (Veh+m, Veh+Gq and Veh+Gi) (P < 0.05, **Supplementary** **Figures 3-6B**), indicating that CNO did not affect the effect of IDPN on motor coordination.

Compared with Veh+m group, the retention time on the rotarod of the Veh+Gq group and Veh+Gi group did not significantly change (P > 0.05, **Supplementary Figures 3-6B**). And compared with TS+m group, the retention time on the rotarod of TS+Gq group and TS+Gi group did not significantly change (P > 0.05, **Supplementary Figures 3-6B**). It showed that chemogenetic activation or inhibition D1R or D2R-containing neurons in the SNpc and dSTR had no significant effect on motor coordination in mice.

The result of grip strength test showed that there was no significant difference between each IDPN-induced TS model group and each vehicle group (P > 0.05, **Supplementary** **Figures 3-6C**), indicating that IDPN had no significant effect on forelimb muscle strength in mice. Meanwhile, after injection of CNO, there was no significant difference in forelimb muscle strength in mice among each group (P > 0.05, **Supplementary Figures 3-6D**), indicating that CNO and chemogenetic activation or inhibition D1R or D2R-containing neurons in the SNpc and dSTR had no significant effect on grip strength in mice.

# Supplementary Figures and Tables

## Supplementary Figures

**
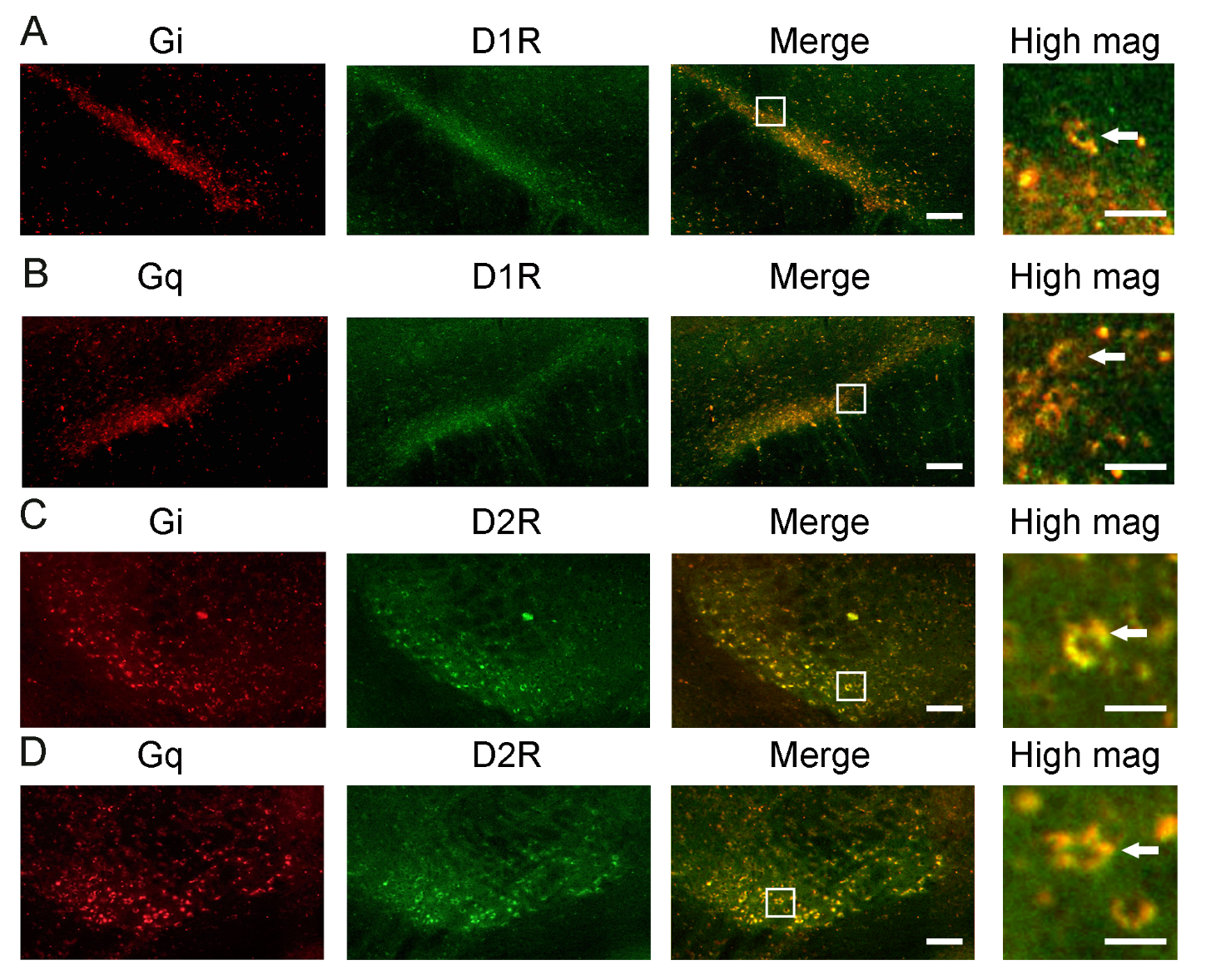
**

**Supplementary Figure 1.** **The co-labeled of hM4Di, hM3Dq and D1R, D2R positive neurons in the SNpc.** (**A**) Co-labeled representative fluorescence images showed the hM4Di (red) and D1R (green) in the SNpc from D1R-cre+Vehicle+Gi+i.p. Saline group. (**B**) Co-labeled representative fluorescence images showed the hM3Dq (red) and D1R (green) in the SNpc from D1R-cre+Vehicle+Gq+i.p. Saline group. (**C**) Co-labeled representative fluorescence images showed the hM4Di (red) and D2R (green) in the SNpc from D2R-cre+Vehicle+Gi+i.p. Saline group. (**D**) Co-labeled representative fluorescence images showed the hM3Dq (red) and D2R (green) in the SNpc from D2R-cre+Vehicle+Gq+i.p. Saline group. n = 6 mice in each group. The white square, a locally enlarged area. White arrows indicate cells that are co-labeled. Scale bar for merge images, 100 μm. Scale bar for high magnification images (high mag), 25 μm.


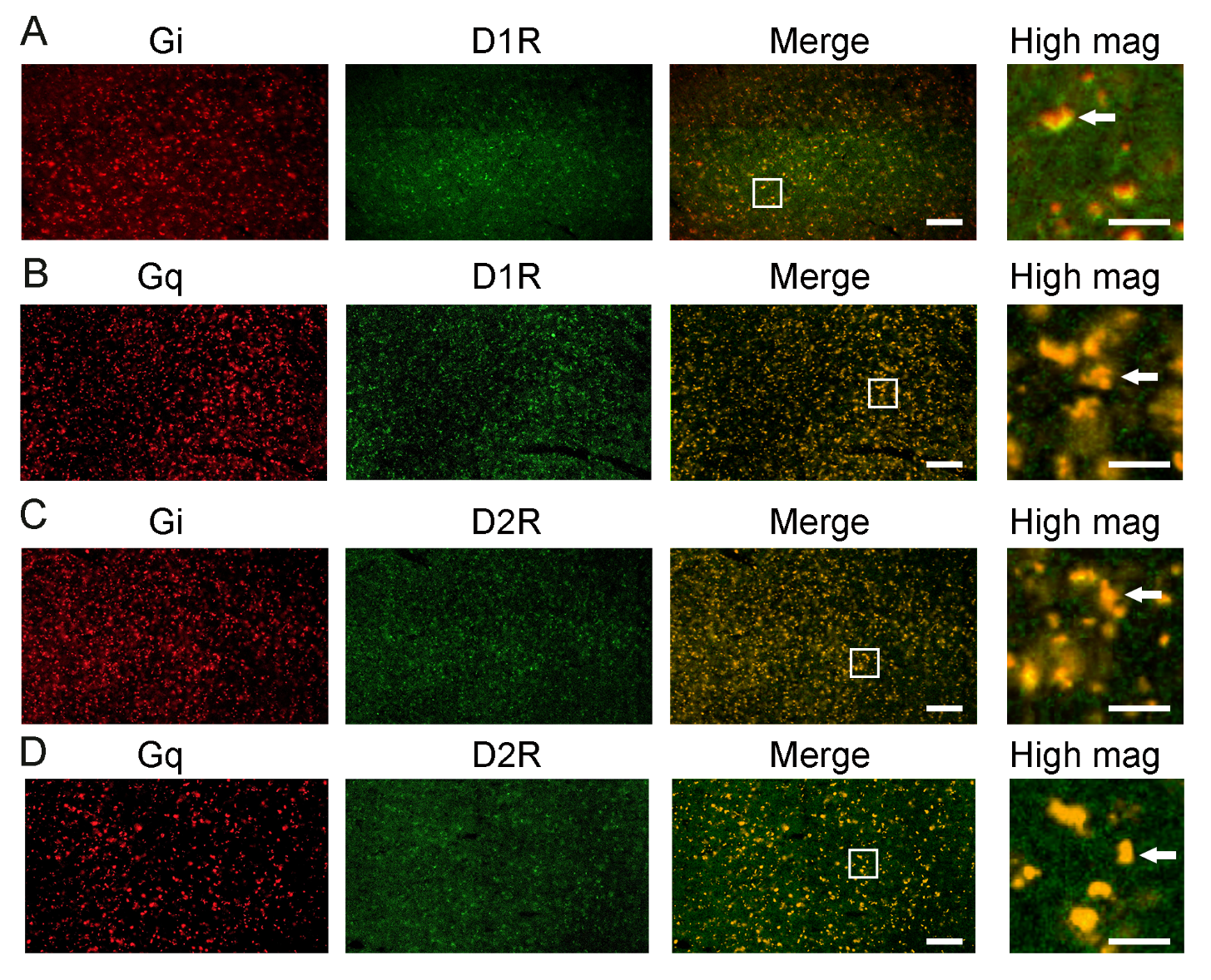


**Supplementary Figure 2. The co-labeled of hM4Di, hM3Dq and D1R, D2R positive neurons in the dSTR.** (**A**) Co-labeled representative fluorescence images showed the hM4Di (red) and D1R (green) in the dSTR from D1R-cre+Vehicle+Gi+i.p. Saline group. (**B**) Co-labeled representative fluorescence images showed the hM3Dq (red) and D1R (green) in the dSTR from D1R-cre+Vehicle+Gq+i.p. Saline group. (**C**) Co-labeled representative fluorescence images showed the hM4Di (red) and D2R (green) in the dSTR from D2R-cre+Vehicle+Gi+i.p. Saline group. (**D**) Co-labeled representative fluorescence images showed the hM3Dq (red) and D2R (green) in the dSTR from D2R-cre+Vehicle+Gq+i.p. Saline group. n = 6 mice in each group. The white square, a locally enlarged area. White arrows indicate cells that are co-labeled. Scale bar for merge images, 100 μm. Scale bar for high magnification images (high mag), 25 μm.


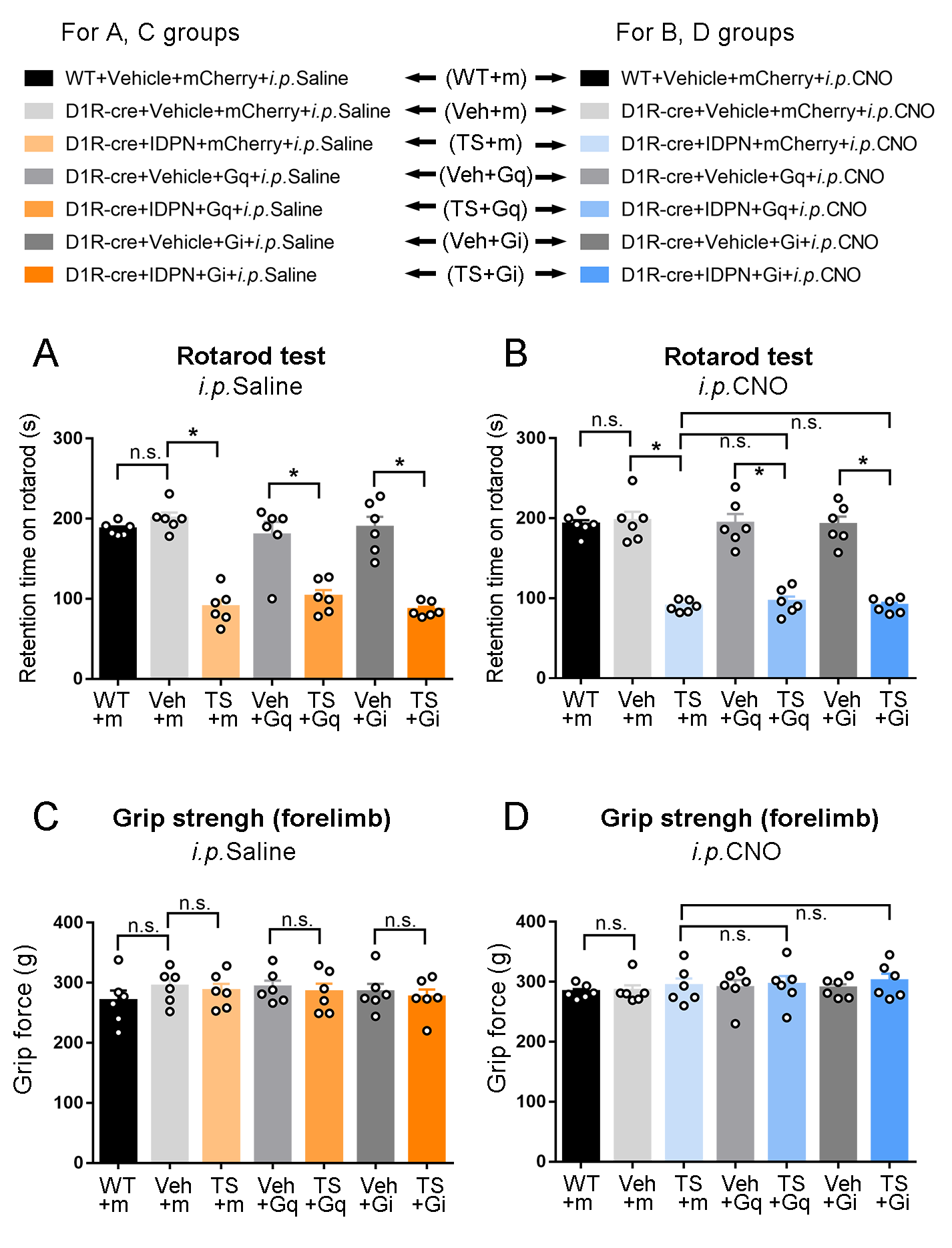


**Supplementary Figure 3. Effects of activation or inhibition of the D1R-containing neurons in the SNpc on motor coordination and forelimb muscle strength in mice.** (**A**) Residence time (s) of mice in the WT+ Vehicle+mCherry+i.p. Saline group (WT+m), D1R-cre+Vehicle+mCherry+i.p. Saline group (Veh+m), D1R-cre+IDPN+mCherry+i.p. Saline group (TS+m), D1R-cre+Vehicle+Gq+i.p. Saline group (Veh+Gq), D1R-cre+IDPN+Gq+i.p. Saline group (TS+Gq), D1R-cre+Vehicle+Gi+i.p. Saline group (Veh+Gi), D1R-cre+IDPN+Gi+i.p. Saline group (TS+Gi) on the rotating rod 30 minutes after injection of saline (i.p. Saline) on day 23. (**B**) Residence time (s) of mice in the WT+ Vehicle+mCherry+i.p. Saline group (WT+m), D1R-cre+Vehicle+mCherry+i.p. Saline group (Veh+m), D1R-cre+IDPN+mCherry+i.p. Saline group (TS+m), D1R-cre+Vehicle+Gq+i.p. Saline group (Veh+Gq), D1R-cre+IDPN+Gq+i.p. Saline group (TS+Gq), D1R-cre+Vehicle+Gi+i.p. Saline group (Veh+Gi), D1R-cre+IDPN+Gi+i.p. Saline group (TS+Gi) on the rotating rod 30 minutes after injection of CNO (i.p. CNO) on day 23. (**C**) Forelimb grip force of mice in each group 30 minutes after injection of saline (i.p. Saline) on day 23. (**D**) Forelimb grip force of mice in each group 30 minutes after injection of CNO (i.p. CNO) on day 23. Data are expressed as mean ± SEM (n = 6 mice in each group), and the black line segment indicates the differences between groups, * represents P<0.05 between marked groups, n.s. represents P＞0.05 between marked groups.


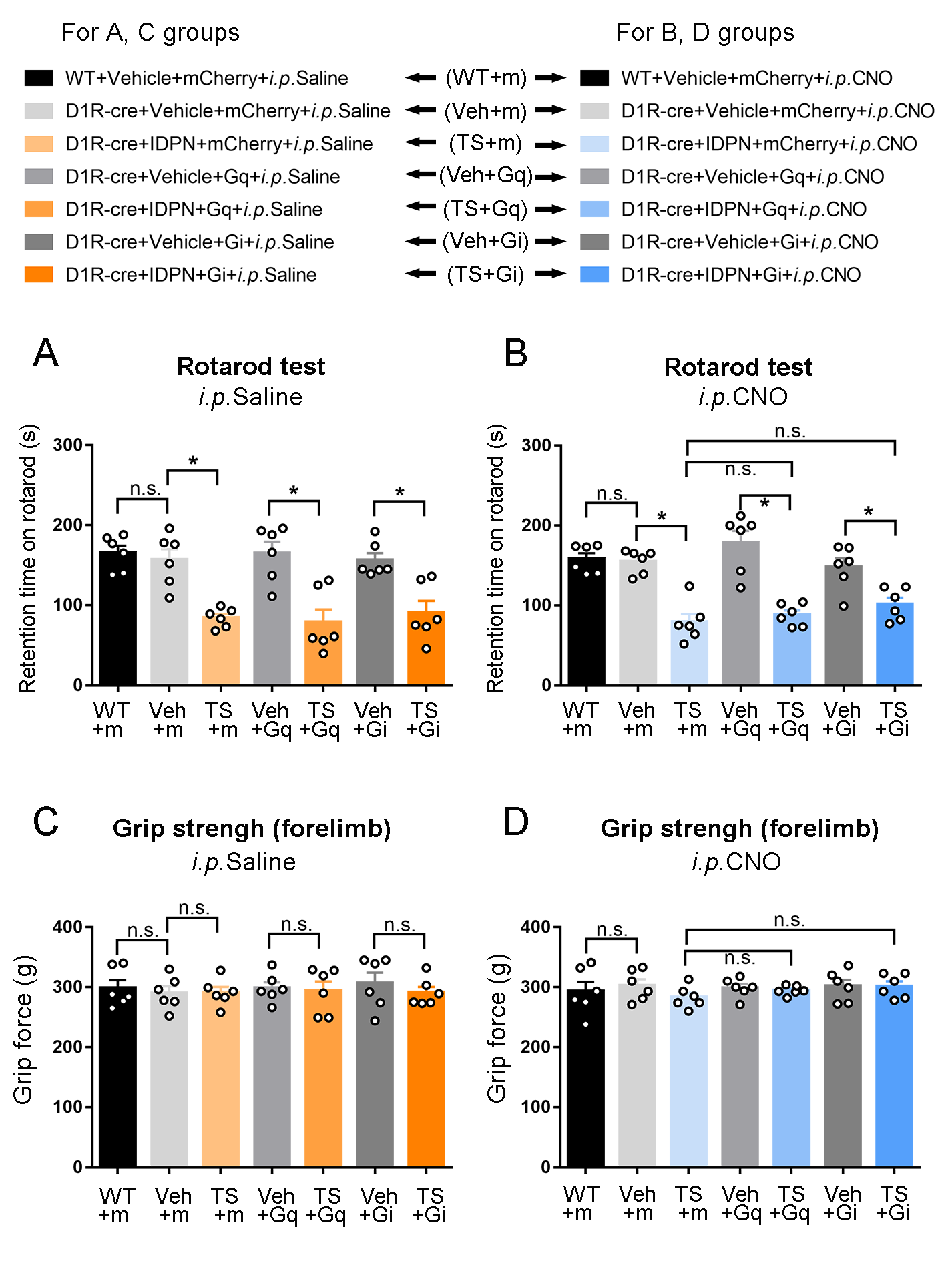


**Supplementary Figure 4. Effects of activation or inhibition of the D1R-containing neurons in the dSTR on motor coordination and forelimb muscle strength in mice.** (**A**) Residence time (s) of mice in the WT+ Vehicle+mCherry+i.p. Saline group (WT+m), D1R-cre+Vehicle+mCherry+i.p. Saline group (Veh+m), D1R-cre+IDPN+mCherry+i.p. Saline group (TS+m), D1R-cre+Vehicle+Gq+i.p. Saline group (Veh+Gq), D1R-cre+IDPN+Gq+i.p. Saline group (TS+Gq), D1R-cre+Vehicle+Gi+i.p. Saline group (Veh+Gi), D1R-cre+IDPN+Gi+i.p. Saline group (TS+Gi) on the rotating rod 30 minutes after injection of saline (i.p. Saline) on day 23. (**B**) Residence time (s) of mice in the WT+ Vehicle+mCherry+i.p. Saline group (WT+m), D1R-cre+Vehicle+mCherry+i.p. Saline group (Veh+m), D1R-cre+IDPN+mCherry+i.p. Saline group (TS+m), D1R-cre+Vehicle+Gq+i.p. Saline group (Veh+Gq), D1R-cre+IDPN+Gq+i.p. Saline group (TS+Gq), D1R-cre+Vehicle+Gi+i.p. Saline group (Veh+Gi), D1R-cre+IDPN+Gi+i.p. Saline group (TS+Gi) on the rotating rod 30 minutes after injection of CNO (i.p. CNO) on day 23. (**C**) Forelimb grip force of mice in each group 30 minutes after injection of saline (i.p. Saline) on day 23. (**D**) Forelimb grip force of mice in each group 30 minutes after injection of CNO (i.p. CNO) on day 23. Data are expressed as mean ± SEM (n = 6 mice in each group), and the black line segment indicates the differences between groups, * represents P<0.05 between marked groups, n.s. represents P＞0.05 between marked groups.


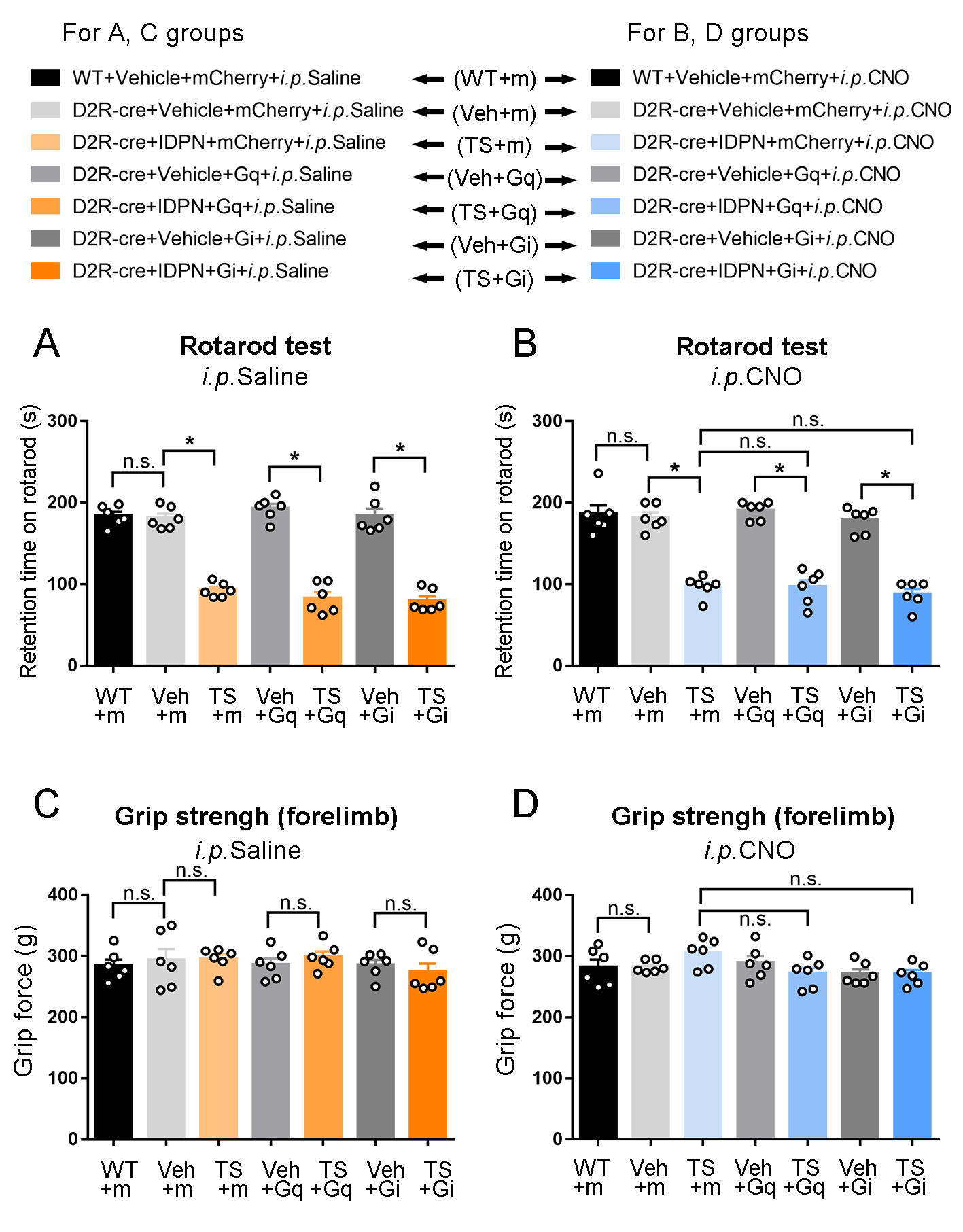


**Supplementary Figure 5. Effects of activation or inhibition of the D2R-containing neurons in the SNpc on motor coordination and forelimb muscle strength in mice.** (**A**) Residence time (s) of mice in the WT+ Vehicle+mCherry+i.p. Saline group (WT+m), D2R-cre+Vehicle+mCherry+i.p. Saline group (Veh+m), D2R-cre+IDPN+mCherry+i.p. Saline group (TS+m), D2R-cre+Vehicle+Gq+i.p. Saline group (Veh+Gq), D2R-cre+IDPN+Gq+i.p. Saline group (TS+Gq), D2R-cre+Vehicle+Gi+i.p. Saline group (Veh+Gi), D2R-cre+IDPN+Gi+i.p. Saline group (TS+Gi) on the rotating rod 30 minutes after injection of saline (i.p. Saline) on day 23. (**B**) Residence time (s) of mice in the WT+ Vehicle+mCherry+i.p. Saline group (WT+m), D2R-cre+Vehicle+mCherry+i.p. Saline group (Veh+m), D2R-cre+IDPN+mCherry+i.p. Saline group (TS+m), D2R-cre+Vehicle+Gq+i.p. Saline group (Veh+Gq), D2R-cre+IDPN+Gq+i.p. Saline group (TS+Gq), D2R-cre+Vehicle+Gi+i.p. Saline group (Veh+Gi), D2R-cre+IDPN+Gi+i.p. Saline group (TS+Gi) on the rotating rod 30 minutes after injection of CNO (i.p. CNO) on day 23. (**C**) Forelimb grip force of mice in each group 30 minutes after injection of saline (i.p. Saline) on day 23. (**D**) Forelimb grip force of mice in each group 30 minutes after injection of CNO (i.p. CNO) on day 23. Data are expressed as mean ± SEM (n = 6 mice in each group), and the black line segment indicates the differences between groups, * represents P<0.05 between marked groups, n.s. represents P＞0.05 between marked groups.


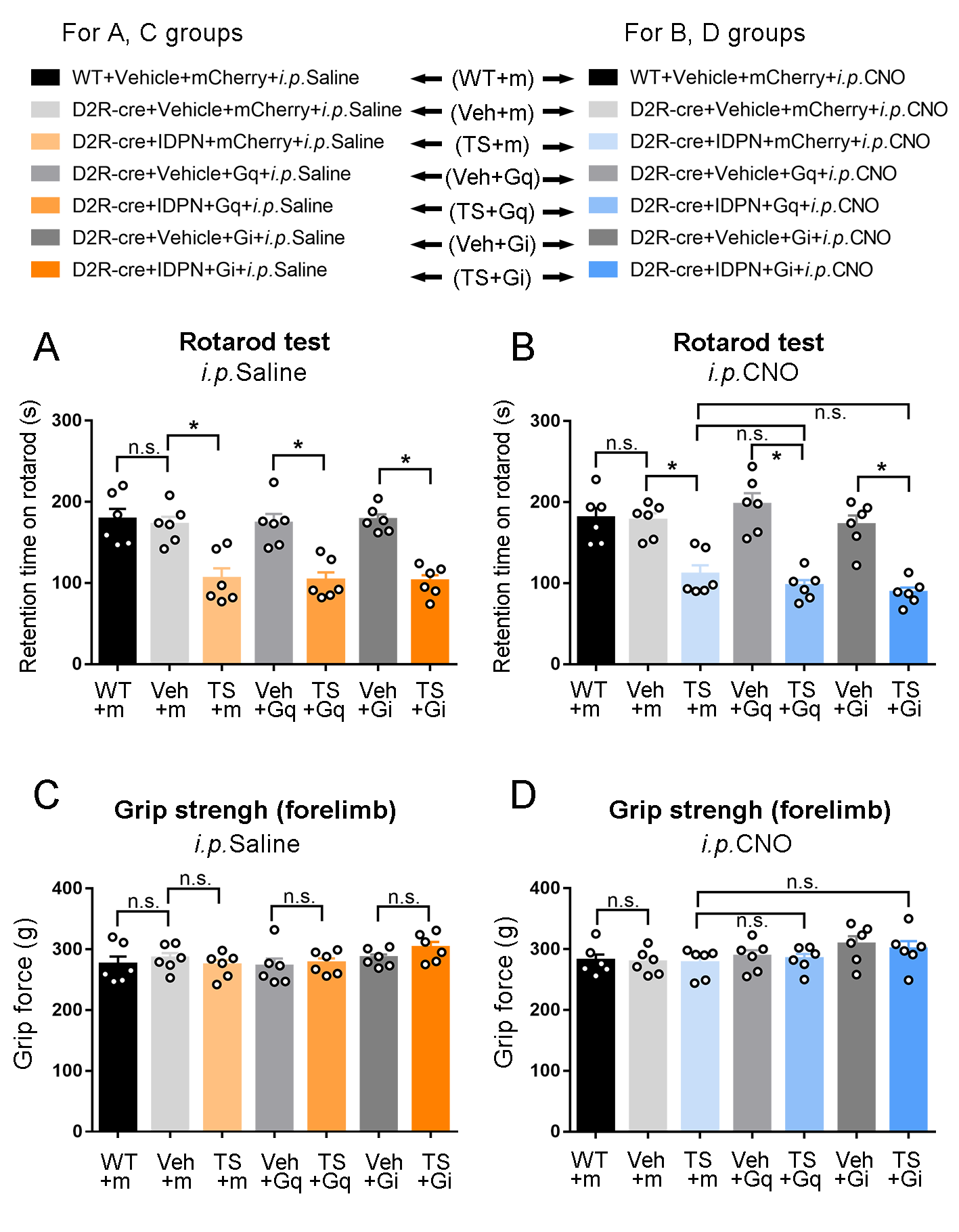


**Supplementary Figure 6. Effects of activation or inhibition of the D2R-containing neurons in the dSTR on motor coordination and forelimb muscle strength in mice.** (**A**) Residence time (s) of mice in the WT+ Vehicle+mCherry+i.p. Saline group (WT+m), D2R-cre+Vehicle+mCherry+i.p. Saline group (Veh+m), D2R-cre+IDPN+mCherry+i.p. Saline group (TS+m), D2R-cre+Vehicle+Gq+i.p. Saline group (Veh+Gq), D2R-cre+IDPN+Gq+i.p. Saline group (TS+Gq), D2R-cre+Vehicle+Gi+i.p. Saline group (Veh+Gi), D2R-cre+IDPN+Gi+i.p. Saline group (TS+Gi) on the rotating rod 30 minutes after injection of saline (i.p. Saline) on day 23. (**B**) Residence time (s) of mice in the WT+ Vehicle+mCherry+i.p. Saline group (WT+m), D2R-cre+Vehicle+mCherry+i.p. Saline group (Veh+m), D2R-cre+IDPN+mCherry+i.p. Saline group (TS+m), D2R-cre+Vehicle+Gq+i.p. Saline group (Veh+Gq), D2R-cre+IDPN+Gq+i.p. Saline group (TS+Gq), D2R-cre+Vehicle+Gi+i.p. Saline group (Veh+Gi), D2R-cre+IDPN+Gi+i.p. Saline group (TS+Gi) on the rotating rod 30 minutes after injection of CNO (i.p. CNO) on day 23. (**C**) Forelimb grip force of mice in each group 30 minutes after injection of saline (i.p. Saline) on day 23. (**D**) Forelimb grip force of mice in each group 30 minutes after injection of CNO (i.p. CNO) on day 23. Data are expressed as mean ± SEM (n = 6 mice in each group), and the black line segment indicates the differences between groups, * represents P<0.05 between marked groups, n.s. represents P＞0.05 between marked groups.

## Supplementary Table

**Supplementary Table 1. Table summarizing the results.**

|  | Behavioral stereotypies | Motor coordination | Grip strength | Total distance in the open field test | Resting time in the open field test |
| --- | --- | --- | --- | --- | --- |
| Chemogenetic inhibition of D1R-containing neurons in the SNpc | | | | | |
| VS Veh+m | — | — | — | — | — |
| VS TS+m | ↓ | — | — | ↓ | ↑ |
| Chemogenetic activation of D1R-containing neurons in the SNpc | | | | | |
| VS Veh+m | — | — | — | — | — |
| VS TS+m | — | — | — | — | — |
| Chemogenetic inhibition of D1R-containing neurons in the dSTR | | | | | |
| VS Veh+m | — | — | — | — | — |
| VS TS+m | ↓ | — | — | ↓ | ↑ |
| Chemogenetic activation of D1R-containing neurons in the dSTR | | | | | |
| VS Veh+m | ↑ | — | — | ↑ | ↓ |
| VS TS+m | — | — | — | — | — |
| Chemogenetic inhibition of D2R-containing neurons in the SNpc | | | | | |
| VS Veh+m | — | — | — | — | — |
| VS TS+m | ↓ | — | — | ↓ | ↑ |
| Chemogenetic activation of D2R-containing neurons in the SNpc | | | | | |
| VS Veh+m | — | — | — | — | — |
| VS TS+m | — | — | — | — | — |
| Chemogenetic inhibition of D2R-containing neurons in the dSTR | | | | | |
| VS Veh+m | — | — | — | — | — |
| VS TS+m | ↓ | — | — | ↓ | ↑ |
| Chemogenetic activation of D2R-containing neurons in the dSTR | | | | | |
| VS Veh+m | — | — | — | — | — |
| VS TS+m | — | — | — | — | — |

^*^— represents no significant change, ↑ represents significant upregulation, ↓ represents significant downregulation.
